# The contraceptive medroxyprogesterone acetate, unlike norethisterone, directly increases R5 HIV-1 infection in human cervical explant tissue at physiologically relevant concentrations

**DOI:** 10.1038/s41598-019-40756-7

**Published:** 2019-03-13

**Authors:** Roslyn M. Ray, Michelle F. Maritz, Chanel Avenant, Michele Tomasicchio, Sigcinile Dlamini, Zephne van der Spuy, Janet P. Hapgood

**Affiliations:** 1 Department of Molecular and Cell Biology, University of Cape Town, Cape Town, South Africa; 2 Centre for Lung Infection and Immunity, Division of Pulmonology and UCT Lung Institute, Department of Medicine, University of Cape Town, Cape Town, South Africa; 3 Department of Obstetrics and Gynaecology, University of Cape Town, Groote Schuur Hospital, Cape Town, South Africa; 4 Institute of Infectious Diseases and Molecular Medicine, University of Cape Town, Cape Town, South Africa

## Abstract

The intramuscular progestin-only injectable contraceptive, depo-medroxyprogesterone acetate (DMPA-IM), is more widely used in Sub-Saharan Africa than another injectable contraceptive, norethisterone enanthate (NET-EN). Epidemiological data show a significant 1.4-fold increased risk of HIV-1 acquisition for DMPA-IM usage, while no such association is shown from limited data for NET-EN. We show that MPA, unlike NET, significantly increases R5-tropic but not X4-tropic HIV-1 replication *ex vivo* in human endocervical and ectocervical explant tissue from pre-menopausal donors, at physiologically relevant doses. Results support a mechanism whereby MPA, unlike NET, acts via the glucocorticoid receptor (GR) to increase HIV-1 replication in cervical tissue by increasing the relative frequency of CD4+ T cells and activated monocytes. We show that MPA, unlike NET, increases mRNA expression of the CD4 HIV-1 receptor and CCR5 but not CXCR4 chemokine receptors, via the GR. However, increased density of CD4 on CD3+ cells was not observed with MPA by flow cytometry of digested tissue. Results suggest that DMPA-IM may increase HIV-1 acquisition *in vivo* at least in part via direct effects on cervical tissue to increase founder R5-tropic HIV-1 replication. Our findings support differential biological mechanisms and disaggregation of DMPA-IM and NET-EN regarding HIV-1 acquisition risk category for use in high risk areas.

## Introduction

Access to safe, affordable and suitable forms of contraception is critical for young women in areas with high HIV-1 acquisition risk and AIDS prevalence. The majority of new HIV infections occur in women and in Sub-Saharan Africa^1,2^. While many different types of contraception are available globally, the most common form in developing countries, where choice is limited, is the 3-monthly 150 mg intramuscular injection of depo-medroxyprogesterone acetate (Depo-Provera or DMPA-IM). Norethisterone enanthate (Nur-Isterate or NET-EN), a 2-monthly 200 mg injection, is less widely used in developing countries. A 30% lower dose (104 mg), 3-monthly subcutaneous injectable contraceptive, DMPA-SC (Sayana**®** Press), with advantages of self-administration, is currently being widely introduced. An estimated 16.5 million women aged 15–49 used Depo-Provera or Nur-Isterate injectable contraceptives in Sub-Saharan Africa in 2014, and these numbers are increasing annually^3^.

Worldwide analysis shows Sub-Saharan Africa is the region with the highest use of DMPA-IM injectable contraception and the highest HIV-1 prevalence^4^. Of great concern is that meta-analyses of epidemiological data suggest a significant 1.4-fold increased risk of HIV-1 acquisition for DMPA-IM users compared to no contraception, although the data may be confounded by behavioural factors^5–7^. No such association is shown for NET-EN compared to no contraception, although those studies are generally underpowered with large confidence intervals, while no information is available for DMPA-SC and HIV-1 acquisition risk^5,6^. Furthermore, limited head-to-head studies suggest a significant 1.3 to 1.4-fold increase in HIV-1 acquisition risk for DMPA-IM compared to NET-EN, although these studies have important limitations^6,8^. In 2017, the World Health Organization modified the Medical Eligibility Criteria (MEC) for Contraceptive use of progestin-only contraceptive injectables, including DMPA-IM, DMPA-SC and NET-EN, to MEC2, and advised that these methods may increase risk of HIV acquisition^9^. To address these concerns with DMPA-IM, a randomised clinical trial (ECHO trial: NCT02550067) assessing HIV-1 acquisition in women using DMPA-IM, relative to levonorgestrel (LNG) implant and copper intrauterine devices (copper-IUD) is currently ongoing. The trial involves 7800 women at several sites in Sub-Saharan Africa in areas at high risk for HIV-1 acquisition, with results expected in 2019^10^. However, this trial will not assess the relative or absolute risk of HIV-1 acquisition of DMPA-IM, DMPA-SC or NET-EN. Determination of the absolute and relative risk factors for HIV-1 acquisition and biological mechanisms for DMPA-IM, DMPA-SC and NET-EN is a critical issue for women’s health, especially in developing countries^11^. Disaggregation of these injectables is highly relevant for choice of contraceptive in these areas, especially given the widespread acceptability and contraceptive efficacy of injectables.

Clinical data suggest several plausible biological mechanisms whereby DMPA-IM may increase HIV-1 acquisition in the female genital tract (FGT)^12^. These include increased frequency of CCR5^+^ T cells in the FGT mucosa^13^, increased expression levels of CCR5 on peripheral and FGT target T cells^13,14^, increased permeability of the FGT^15^ and alterations in levels of select secreted immunomodulators^16–24^. Many of these studies are consistent with data from animal models using DMPA doses resulting in similar MPA serum concentrations to those of human DMPA-IM users^15,25–29^ as well as with several *ex vivo* studies^30–33^.

We have previously shown that MPA and NET have very different biological effects due to differences in their glucocorticoid-like properties. MPA binds to the glucocorticoid receptor (GR) with a relatively high affinity and acts like a full to partial GR agonist, depending on cellular context, while NET exhibits almost no GR activity^34,35^. The concentration required for half maximal activity or EC_50_ for MPA regulation of gene expression via the GR in *ex vivo* cell models is in the range of about 1–100 nM and varies in a cell- and gene-specific manner^32,36–41^, while NET shows no response via the GR^32,36–38^. This is well within the range of peak serum levels (C_max_) of MPA in DMPA-IM users. These vary widely between individuals and between studies from 3 to 100 nM^12,42–55^, while plateau levels of about 2.6 nM are maintained for the rest of the 3 months^44,46,50–52,56–58^. When evaluated separately, the reported C_max_ values differ greatly between DMPA-SC and DMPA-IM, with an average C_max_ value for DMPA-SC of about 3.3 nM^59–61^, while no significant differences have been detected in the mean serum trough levels from one head-to-head study^62^. However, whether a difference exists between DMPA-IM and DMPA-SC C_max_ values^11^ remains to be determined, since these have not been evaluated in head-to-head studies. The C_max_ values reported for NET-EN of 10–50 nM^12,46,54,63,64^ are in a similar range to those reported for DMPA-IM.

Based on the above, as well as the ubiquitous expression of the GR, unlike the progesterone receptor (PR) or androgen receptor (AR)^34^, MPA would be expected to exert widely different biological effects on multiple physiological processes via the GR, unlike NET. We have recently shown that physiologically relevant concentrations of MPA, unlike NET, increases HIV-1 infection *ex vivo* in human peripheral blood mononuclear cells (PBMCs) via a GR-dependent mechanism^65^. We found that this involves, at least in part, increased expression of the CCR5 HIV-1 coreceptor on target T-lymphocytes^65^. MPA, unlike NET, also increased activation of T-cells and increased the CD4/CD8 ratio *ex vivo* in PBMCs^65^. However, it is not known whether MPA or NET have direct or different effects on HIV-1 pathogenesis in human genital tract tissue. In this study, we investigated our hypothesis that MPA will have different effects on HIV-1 replication in explant tissue compared to NET due to its inherent different molecular properties and actions via the GR. We show for the first time that MPA, unlike equimolar NET, dose-dependently increases replication of founder R5 HIV-1 virus and expression of CCR5 mRNA, via the GR, at concentrations within the range of those found in the serum of DMPA-IM and NET-EN users.

## Methods and Materials

### Ethics

Permission to perform these studies was granted by the Human Research Ethics Committee of the Faculty of Health Sciences of the University of Cape Town (approval number: HREC 210/2011) and all procedures were approved and carried out according to the established guidelines. Women who were undergoing appropriate gynaecological surgery for benign indications were informed about the study and those willing to donate a small sample of their post-surgery specimen completed a written and signed informed consent form. Recruitment was undertaken by a member of the clinical research team who was not involved in the patient’s clinical care. All possible participants were assured that their decision about whether or not to contribute to the study would have no impact on their clinical management and follow up.

### Compounds, antibodies and plasmids

(11β,16α)-9-fluoro-11,17,21- trihydroxy-16-methylpregna-1,4-diene-3,20-dione (dexamethasone; DEX, D4902), 6α-methyl-17α-hydroxy-progesterone acetate (medroxyprogesterone acetate; MPA, M1629), 4-pregnene-3,20-dione (progesterone; P4, P0130), 17α-ethynyl-19- nortestosterone (norethisterone; NET, N4128), and 11β-(4-dimethylamino) phenyl-17β-hydroxy-17-(1propynyl) estra-4,9-dien-3-one (Mifepristone; RU486, M8046) were purchased from Sigma-Aldrich, South Africa. Interleukin 2 (IL2) and Maraviroc (MVC) were obtained through the AIDS Research and Reference Reagent Program, Division of AIDS, NIAID, NIH. 3-(4,5-dimethylthiazol-2-yl)-2,5-diphenyltetrazolium bromide (MTT, M5655) and lamivudine (3TC) were purchased from Sigma-Aldrich, South Africa. Antibodies to GR (H-300, sc-8992), and GAPDH (0411; sc-47724) were obtained from Santa Cruz Biotechnology, USA. Secondary antibodies for primary detection were purchased from Santa Cruz Biotechnology, USA, and include anti-mouse (sc-2005) and anti-rabbit (sc-2313). Anti-CD3 fluorescein isothiocyanate (FITC), (300440), anti-CD4 phycoerythrin-Dazzle 594 (PE-Dazzle 594) (357412), anti-CD8 PE/Cy5 (300910), anti-CD69 PE/Cy7 (310912), anti-CCR5 allophycocyanin (APC) (359122) and ZOMBIE NIR (423113) were purchased from Biolegend (USA). A X4-tropic IMC, pNL4-3, was obtained through the AIDS Research and Reference Reagent Program, Division of AIDS, NIAID, NIH, from Dr. Malcolm Martin^66^, and named HIV-1_pNL4.3_ in this study. An R5 infectious molecular clone that had a luciferase gene inserted adjacent to the *env* gene in the HIV-1 NL4-3 backbone known as NL–LucR.T2A–BaL.ecto, was a kind gift from Dr. Christina Ochsenbauer^67^, and known as HIV-1_BaL_Renilla_ in this study.

### Cell Culture

Human embryonic kidney cells (HEK293T) were purchased from America Type Culture Collection (ATCC, USA). Human cervical TZM-bl cells, were procured from the NIH AIDS Reagent Program, Division of AIDS, NIAID, NIH from Dr. John C. Kappes, Dr. Xiaoyun Wu and Tranzyme Inc. (ARP, NIH, USA). Cells were cultured in 75 cm^2^ flasks (Greiner Bio-one International, Austria) in Dulbecco’s modified Eagle’s medium [(DMEM) (Sigma-Aldrich, South Africa) supplemented with 1 mM sodium pyruvate (Sigma-Aldrich, South Africa), 44 mM sodium bicarbonate (Sigma-Aldrich, South Africa), 10% (v/v) foetal bovine serum (Thermo Scientific, South Africa) 100 IU/mL penicillin and 100 mg/mL streptomycin (P4333, Sigma-Aldrich, South Africa); full DMEM]. All cells were maintained at 37 **°**C in a water jacket incubator (90% humidity and 5% CO_2_). Cells were passaged twice a week with 0.25% (w/v) trypsin/ 0.1% (w/v) EDTA in PBS (Highveld Biological, South Africa). Trypsinisation was terminated with neutralisation medium (full DMEM). All cells were routinely tested and found to be mycoplasma free.

### Virus Propagation

Initial viral stocks were prepared as previously described^68^ with a few modifications. HEK293T cells were seeded at a density of 4 × 10^6^ cells/10 cm^2^ plate in full DMEM supplemented with 25 mM HEPES buffer (Lonza, Germany) at 37 **°**C in a water jacket incubator (90% humidity and 5% CO_2_). The next day, media was replaced, and cells were transfected with 12 μg HIV-1_pNL4.3_, HIV-1_BaL-Renilla_ or a control (DMEM) using X-tremeGENE 9 DNA transfection reagent (Roche Applied Science, South Africa) according to the manufacturer’s specifications. Cells were incubated for 48 hr at 37 **°**C, the medium was passed through a 0.22 μM filter and charcoal-stripped (c-s) FCS (Thermo Scientific, USA) was added to a final concentration of 12.5%. The viral stocks were aliquoted and stored at −80 **°**C until use. Virus titres were determined using the TZM-bl assay as previously described^67^. Cells were harvested 72 hr later with 120 μL Bright-Glo luciferase lysis buffer (Promega, USA). Fluorescence was determined on a luminometer (Modulus Microplate, Promega, USA), where relative light units were measured for each well. The titre of the virus stock was determined using the Reed Muench method and expressed as log infectious units (IU)/mL^69^.

### Explant Culture and Infection Experiments

Cervical tissue was obtained from HIV-1 negative, pre-menopausal women, not on contraception, with a normal pap smear and undergoing hysterectomies for benign reasons, after informed consent. Fresh tissue was supplied from two sites in the Western Cape, South Africa, namely Groote Schuur Hospital and Tygerberg Hospital. Inclusion criteria and donor consent were confirmed prior to the operation. Blood samples were collected and sent to the National Health Laboratory Services (NHLS, Groote Schuur Hospital, South Africa) for serum antibody testing of HIV-1 and HSV 1/2 status. The majority of the samples were positive for HSV-1 and negative for HSV-2. Additionally, serum levels of luteinising hormone (LH), follicle stimulating hormone (FSH), progesterone (P4) and estrogen (E2) were obtained from the majority of donors from blood samples and used to define the phase of the menstrual cycle, together with information on the clerking sheets. Menstrual cycle was determined using the guidelines given by the NHLS (South Africa) (see Supplementary Table S1). Supplementary Table S2 summarizes donor information including HSV status, hormone levels, age, stage of menstrual cycle and reasons for surgery. When comparing matched conditions performed on the same donor, differences in donor hormone levels are unlikely to account for differences in results between conditions. We did not detect any statistically significant differences between endogenous hormone levels of donor samples used for non-matched incubations (see Supplementary Fig. S1). We routinely received more ectocervical tissue than endocervical tissue, so experiments on endocervical explants were more limited than for ectocervical explants.

Cervical tissue was processed as previously described^70^, between 1 to 3 hr post operation. Excess underlying stromal tissue was removed from the epithelial layer of the ectocervical and endocervical tissue. The epithelial layer was then diced into 3 mm^3^ explant pieces, which were randomly placed into separate wells of 96 well round-bottomed plates. Non-polarised explants were cultured in 200 μL RPMI (Lonza, Switzerland) supplemented with 10% (*v/v*) c-s FCS, 2 mM L-glutamine (Sigma-Aldrich, South Africa), 10 μg/mL Fungizone (Sigma-Aldrich, South Africa), 10 U/mL IL-2, 100 IU/mL penicillin and 100 mg/mL streptomycin (Sigma-Aldrich, South Africa) and incubated at 37 **°**C in a water jacket incubator (90% humidity and 5% CO_2_).

Explants were treated in triplicate or quadruplicate with vehicle control (0.1% [*v/v*] EtOH) or ligands in parallel, at concentrations indicated in the Figures, for 48 hr. Researchers have reported using from 3 to 16 tissue blocks per condition^71,72^. Our choice of block number was based on limited fresh tissue availability and a need to perform matched incubations for different conditions for each donor tissue. For infection, explants were exposed to either 10 000 IU/mL HIV-1_BaL-Renilla_ or HIV-1_pNL4.3_ (in the presence of hormone) for 2 hr at 37 °C in a water jacket incubator. Explants were then washed 4 X with 1 X PBS (containing 1% [*v/v*] cs-FCS) and transferred back to individual wells of a 96 well round-bottomed plate. Explants were incubated in full media containing the appropriate concentration of ligand for a further 10 days post infection. Half-media was exchanged at days 3, 5 and 7 post infection, and replaced with media containing 100 nM of the appropriate ligand for each treatment group for all explant experiments. Media were harvested at days 0, 3, 5, 7 and 10 post infection were aliquoted into flat-bottomed 96 well plates (Griener, Germany) and incubated with 5% (*v/v*) Empigen® (B13 Detergent, 45165, Sigma-Aldrich, South Africa) in 1 X PBS for an hour at room temperature, to ensure viral inactivation. Culture supernatants were spun at 1000 × g for 5 min to remove cellular debris and aliquoted into cryovials and stored at −80 **°**C. Culture supernatants were subsequently assayed for HIV-1 p24_Gag_ release using a p24 ELISA assay (Innotest, Innogenetics, Belgium), as well as in some cases being used to measure soluble mediators or in add-back assays. p24 concentrations were calculated using the equation generated by the standard curve and p24 accumulation curves (pg/mL) was determined. Experiments with an ARV inhibitor showed that 3TC decreased the relative accumulated p24 levels, consistent with productive infection (see Supplementary Fig. S2). Residual activity in the presence of 3TC could possibly be due to some inactivation of the ARV due to metabolism in tissue^73^ or the passive release of HIV or p24 Gag into tissue culture supernatant. Some donor samples showed increased relative p24 accumulation continuously from day zero to day 10, while others did not exhibit an increase in p24 accumulation beyond day 5. Donor samples that exhibited no increase in p24 accumulation after day 3, indicative of unsuccessful infection, were excluded from the analysis (about 20% of experiments). As infectivity varied between donors, pooled data was expressed as relative infection relative to vehicle control at day 3 post infection (EtOH) set to 1. Viability of tissue was assessed either by MTT at the time of processing, 48 hr pre-infection and at 10 days post-infection (data not shown) or the Zombie Cell viability stain 48 hr and 7 days after treatment. It is possible that the use of fresh tissue and the presence of IL2^74^ in our culture medium enhanced cell viability in our experiments.

### TZM-bl Infection and Add-back Reporter Assay

TZM-bls were seeded at a concentration of 1 × 10^5^ cells/mL in a 96-well flat-bottomed culture plate in full DMEM. The following day the TZM-bls were either stimulated with hormone or maraviroc (MVC) for 24 hr in triplicate or supernatants from ectocervical explants, which were diluted 1:10 in DMEM medium, as previously described^75^, with a few modifications. Supernatants were harvested from ectocervical explants that had been stimulated with 100 nM of either MPA, NET, MVC, or with vehicle controls in quadruplicate (0.1% [*v/v*] EtOH or 0.1% [*v/v*] DMSO). Supernatants of treated ectocervical explants were pooled and subsequently centrifuged at 1200 × *g* for 5 minutes at room temperature to pellet cellular debris. As a control to compare the direct effects of MPA and NET on HIV-1 replication, 100 nM MPA, NET or a vehicle control (0.1% EtOH) was incubated in full RPMI media in a 24 well culture dish for 48 hr. Supernatants were collected and stored at −80 °C until use. After 24 hr incubation with either hormone or explant supernatant, cells were infected with 20 IU/mL HIV-1_BaL_Renilla_. Cells were harvested 48 hr later with Bright-Glo luciferase lysis buffer (Promega, USA). Fluorescence was determined on a luminometer (Modulus Microplate, Promega, USA), where relative light units were measured for each well. Viability was measured using the MTT assay and measured on a spectrophotometer (Thermo Scientific, USA) at 595 nm. Luciferase readings were expressed over MTT (RLU/MTT). Relative infection was calculated by setting the vehicle control (EtOH) to 100% relative infection.

### RNA Isolation and Real Time Quantitative PCR (qPCR)

For RNA isolation from explants, tissue was placed into 800 μL Qiazol ® in a 2 mL cryovial tubes (Nunc, Germany). Samples were homogenized on ice using a hand-held homogeniser (TissueRuptor®, Qiagen, Netherlands) with disposable probes (TissueRuptor Probes, Qiagen, Netherlands), for 20 second pulses, for up to 1 minute. Homogenates were transferred to new microfuge tubes and incubated for 5 minutes at room temperature. Samples were subsequently centrifuged at 12 000 × g for 10 minutes at 4 °C and RNA was isolated using the RNeasy Tissue MicroArray Kit (Qiagen, Netherlands), with an on-column DNAse treatment. RNA quality was confirmed by agarose gel electrophoresis and only RNA with 260/230 and 260/280 ratios of >1.8 was used.

Total RNA (250 ng) was reverse-transcribed using the Transcriptor First Strand Synthesis cDNA kit (Roche Applied Science, South Africa) according to the manufacturer’s instructions. cDNA samples were stored at −80 **°**C until use in subsequent real time qPCR reactions. Real-time quantitative PCR (qPCR) was performed using the Bioline SensiMix™ SYBR® no ROX kit (QT650-05, Bioline USA) on a RotorGene 3000 (Qiagen, Netherlands) real time qPCR machine, according to manufacturer’s instructions. The steroid receptor primers and amplification profiles were as previously established^76^. CCR5 and CXCR4 were amplified with the following primer pairs: CCR5 - 5′ TGGACCAAGCTATGCAGGTG 3′ and 5′ CGTGTCACAAGCCCACAGAT 3′. CXCR4: - 5′GAAATGGGCTCAGGGGACTAT 3′ and 5′ TTCAGCCAACAGCTTCCTTGG 3′ with a Ta of 55 °C and 60 °C respectively. CD4 primers and amplification profile were previously established^77^, while for GAPDH the primers were as described by Verhoog *et al*. (2011)^78^. Relative transcript levels were determined using the established methods^79^, with vehicle control set to 1.

### Flow Cytometry

Flow cytometry was performed as previously described^76^ and tissue was digested as described in^80^ with a few modifications. Ectocervical explant tissues were diced as described above but into smaller 0.5 mm^3^ pieces and six explant pieces per condition were placed into a volume of 500 uL of full RPMI media in separate wells of a 24-well plate in the presence of hormones and incubated for 48 hr or 7 days in a water jacket incubator. After 48 hr or 7 days, media was harvested and tissue pieces were washed with 1% PBS containing 1% c-s FCS (1% c-s FCSPBS). Tissue pieces (squares/blocks) were placed into a 1 mL eppendorf tube and digested with 5 µg/mL collagenase II (Cat no: 17101015, Thermo Fisher, USA) in 1 mL of complete Hank Balanced Salt Solution (HBSS) with Calcium and Magnesium (Lonza, Germany) for 45 min on a rotator at 37 °C in a water jacket incubator. Digestion for 25 or 45 min did not change total amount of cells, or the frequency of cells types investigated. Digested tissue was passed through a 70 µM cell strainer (Corning, USA) and washed two times with full RPMI media and a third time with 1% c-s FCSPBS. Filtered cells were collected and pelleted at 400 × *g* (ThermoScientific, swing-bucket centrifuge). Cells were washed twice with 1% c-s FCSPBS, followed by staining in PBS (in the absence of protein) with the appropriate antibodies. Explants were stained with Zombie NIR, anti-CD3 FITC, anti-CD4 PE-DAZZLE 594, anti-CD8 PE/Cy5, anti-CD69 PE/Cy7 and anti-CCR5 PE antibodies (Biolegend, USA), at room temperature for 15 minutes in the dark. After staining, explants were washed with 1% c-s FCSPBS and resuspended in 1 X Cell Fix solution (Becton-Dickinson, USA). Samples were acquired using a LSRII Becton-Dickinson flow cytometer (Becton-Dickinson, USA) and analysed using Flow Jo software (version 10.1, Treestar, Inc, Ashland, Ore). Only the single cellular population were analysed. Dead cells were excluded from the scatter plots prior to analysis and negative gates were set using minus fluorescence one (MFO) controls. Results are represented as either frequency (as a percentage of total) or expression (median fluorescence intensity or MFI]). Relative fold change in frequency or expression levels was calculated by setting vehicle control (EtOH) expression to 1.

### Determination of protein levels of soluble mediators

Supernatants were collected from primary ectocervical explants into 2 mL cryovial tubes (Nunc, Germany) and stored at −80 °C for future use. Choice of soluble mediators for analysis was based on those we deemed likely to be regulated by MPA as indicated from the literature^17–20,24,81^. We designed a panel for screening by luminex for those available, and by ELISA for those not available by luminex. For luminex analysis, cytokine and chemokine secretion was measured using a Millipore Milliplex MAGPLEX-60 (Merck, Germany), according to the manufacturer’s instructions. Plate were read using the Luminex 200 pro (Bio-Rad, Germany) at the SUN Immunology Research Group, University of Stellenbosch (South Africa) that had been validated and calibrated prior to the analysis. Analytes included: IL-1RA, IL6, IL8, Eotaxin, MCP-1, and RANTES. Levels of some soluble mediators including HBD-2, HBD-3, IFN-γ (ABTS ELISA development Kit, Peprotech, USA) fell below the detection limit (10 pg/mL) in our samples and hence were not determined. Sample concentrations were determined using the equation generated for each individual cytokine or chemokine. Relative fold change expression was determined by setting the vehicle control (vehicle, EtOH) to 1. For ELISAs, IL-8, IL-6 and SLP1 secretion was measured by the DuoSet ELISA, according to the manufacturer’s instructions (R&D Systems, USA).

### Statistical Analysis

Results were analysed using GraphPad PRISM (version 6) software (La Jolla California, USA). Data were tested for normalcy before parametric tests were performed using the D’Agostino-Pearson omnibus normality test for large data sets and the Kolmogorov-Smirnov test with Dallal-Wilkonson-Lille for P value for small data sets (n < 6). For the HIV-1 replication experiments in the cervical explants, a repeated measures’ two-way ANOVA was performed, with a post-hoc Tukey test or a post-hoc Sidak’s test to assess the significance between the treatments at each time point assayed. In order to determine the accumulation of p24 over the time course assayed, we used the area under the curve (AUC) method to calculate total p24 accumulated over 5, 7 and 10 days post infection, and normalized these values to the vehicle control set to 1. Where samples were treated with ligands at one time point, a one-way ANOVA, with either a Dunnet’s or Tukey’s post-test comparing each group to control or each group to each other, was performed. For comparison between two samples, an unpaired two-tailed student’s t-test was performed. For data that was non-parametric, a Kruskal-Wallis ANOVA with a Dunn’s multiple comparisons test was performed when comparing samples to each other. For non-parametric data compared to control, a Wilcoxon Signed Rank test was performed, with the hypothetical median set to the control value of 1. Additionally, when two groups were compared to each other, a non-parametric Mann-Whitney test was performed. Data were expressed as mean ± SEM on histograms or mean + SEM on XY scatter charts, with n values given in each Figure legend. Where statistical significance of difference was obtained relative to a single control statistical significance is denoted by *, **, *** or **** to indicate p < 0.05, p < 0.01, p < 0.001, or p < 0.0001 respectively. Where statistical significance of difference between two values was obtained, this is indicated with lines showing the sample sets which were significantly different from each other.

## Results

To investigate whether MPA directly increases HIV-1 replication at key sites in the lower FGT, fresh non-polarized ectocervical or endocervical tissue explants from HIV-1 negative, pre-menopausal donors, not on contraception, were pre-treated for 48 hr in parallel with vehicle, and equimolar concentrations of MPA or NET. Thereafter they were infected with infectious molecular clones (IMCs) of either X4-tropic (HIV-1_pNL4.3_) or R5-tropic (HIV-1_BaL_Renilla_) virus and viral p24 levels were monitored at days 3, 5, 7 and 10, in the presence of vehicle, MPA or NET.

### MPA increases R5-tropic HIV-1 replication in cervical explants at peak serum levels of DMPA-IM users

**Figure 1:**
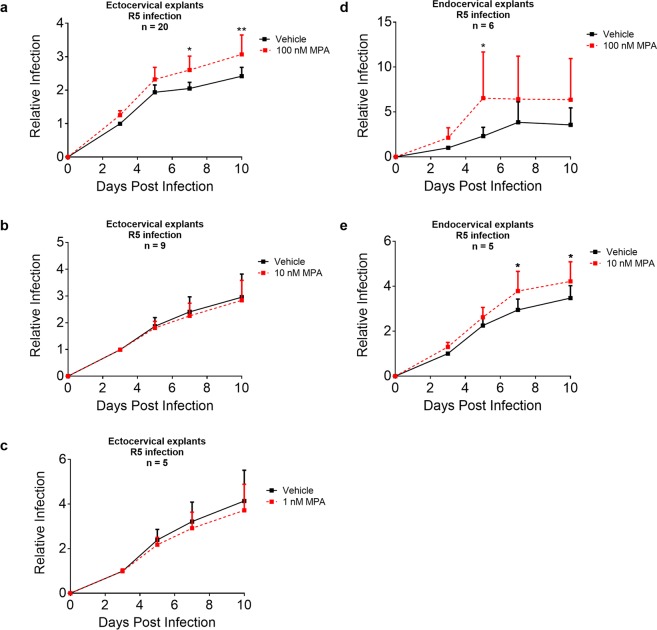
MPA increases R5-tropic HIV-1 replication in cervical explants in a dose-dependent manner and at peak serum levels of DMPA-IM users. (**a**–**c**) Ectocervical or (**d**,**e**) endocervical explants for each donor were treated in parallel with vehicle (EtOH [0.1% v/v]) or 1, 10 or 100 nM MPA for 48 hr after which tissue was infected. washed and left for varying times in the presence of vehicle or the different concentrations of MPA. Media were collected at 3, 5, 7 and 10 days post infection and assayed for p24 levels by ELISA. Infection with R5 HIV-1_BaL-Renilla_ was measured and relative infection was plotted relative to day 3 vehicle set to 1. Relative infection for pooled data was used to compare the fold-change in infection for matched incubations with vehicle or MPA, since the absolute infection levels as measured by p24 assays at day 3 varied in a donor-dependent manner. Each condition was performed at least in triplicate. For data set (**a**), seven of these were performed as independent donor experiments comparing only MPA vs vehicle in parallel. An additional 10 experiments were performed independently with MPA vs NET vs vehicle, used in Fig. 2a, and the MPA vs vehicle data only from these 10 data sets were also used in (**a**). MPA vs vehicle data from three experiments shown in Fig. 3b, were also used in (**a**), making the total for n = 20 independent donor data sets shown in (**a**). Results of b and c are representative of (**b**) nine and (**c**) five independent donor experiments. (**d**) MPA vs vehicle data are from six experiments done in parallel with NET, also shown in Fig. 2b (**e**). Results are shown from 5 independent donor experiments. XY plots are representative of mean + SEM. Statistical significance was determined by comparing MPA to the vehicle control using a repeated measures two-way ANOVA with a post hoc Sidak’s test, with *, **, *** and **** denoting p < 0.05, p < 0.01, p < 0.001 and p < 0.0001, respectively. Detailed statistical analysis is shown in Supplementary Tables S3 and S4. All data sets except one had all data time points. For (**a**) one of the donors (49) experiments was missing a day 5 value and was therefore excluded from the statistical analysis, although it is included in the final Figure.

Incubation of ectocervical explant tissue with 100 nM MPA significantly increased R5-tropic HIV-1 replication compared to vehicle in matched donor incubations by 1.2 to 1.3-fold after 7–10 days (Fig. 1a). Dose response analysis revealed no significant effects with 10 or 1 nM MPA for any of the time points up to 10 days (Fig. 1b,c). Incubation of endocervical explant tissue with 100 nM MPA also significantly increased R5-tropic HIV-1 replication compared to vehicle in matched donor incubations (Fig. 1d) by about 3-fold after 5 days, with no significant changes at later time points (Fig. 1d). However, 10 nM MPA significantly increased R5-tropic HIV-1 replication in endocervical explant tissue compared to vehicle in matched donor incubations by about 1.2- fold after 7 and 10 days (Fig. 1e). For detailed statistical outputs for each data set at each time point see Supplementary Tables S3 and S4. For raw p24 accumulation curves (pg/mL) for select individual donors see Supplementary Fig. S3. An alternative method of analysis by calculating the AUC showed similar results, as illustrated for ectocervical explants incubated with 100 nM MPA (see Supplementary Fig. S4). Supplementary Figs S3 and S4 show that some donors exhibited much greater responses than others to MPA, suggesting donor-specific effects. The relatively small donor numbers did not allow correlation with any extrinsic donor characteristics, including menstrual cycle phase (see Supplementary Table S2). Taken together, the data show that MPA increases HIV-1 replication under our conditions in both ectocervical tissue and endocervical tissue in a dose-dependent manner and at concentrations detected in the serum of DMPA-IM users.

### MPA unlike NET increases HIV-1 replication in cervical explant tissue

**Figure 2:**
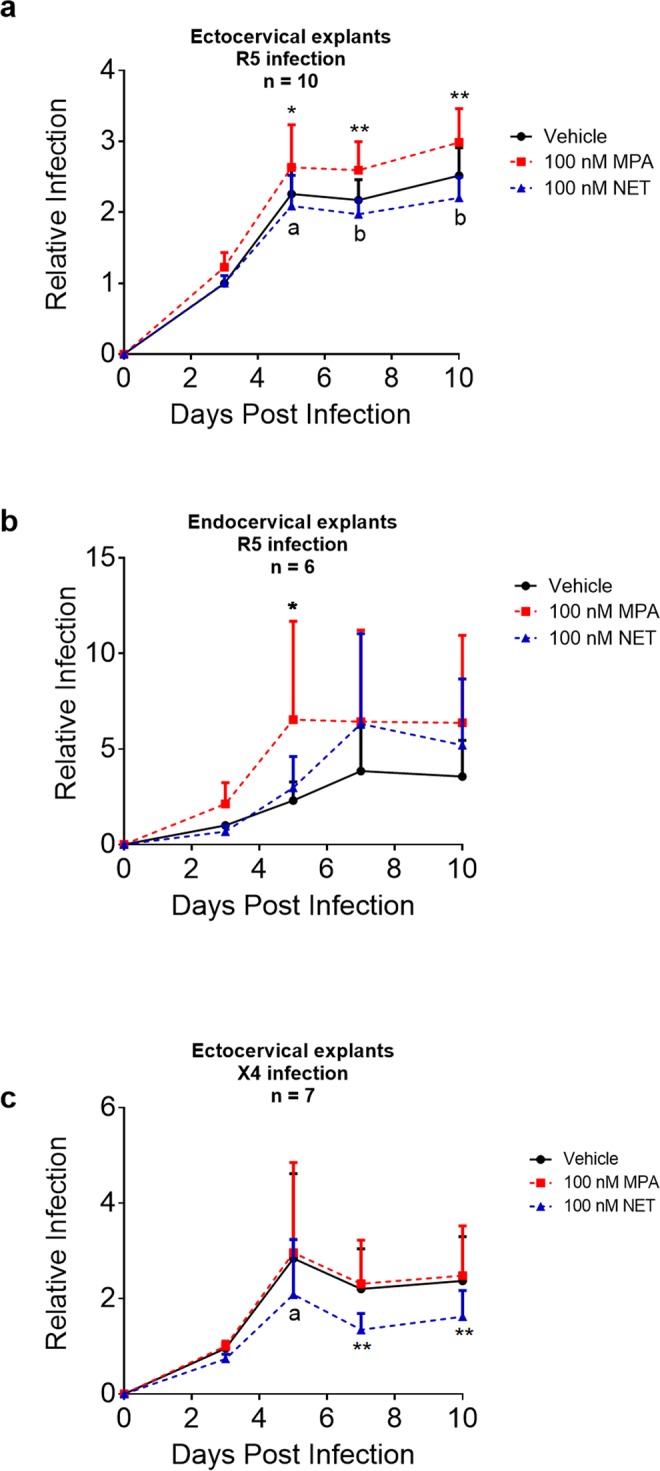
MPA unlike NET increases R5- but not X4-tropic HIV-1 replication in cervical explant tissue. Explants were pre-treated in parallel with vehicle (EtOH [0.1% v/v]) or 100 nM MPA or 100 nM NET for 48 hr after which tissue was infected, washed and incubated for varying times in the presence of vehicle, 100 nM MPA or 100 nM NET. Media were collected at 3, 5, 7 and 10 days post infection and assayed for p24 levels by ELISA. a-c show the relative infection levels at time points 0, 3, 5, 7 and 10 days post infection with either (**a**,**b**) HIV-1_BaL-Renilla_ or (**c**) HIV-1_PNL4.3_ with day 3 vehicle control (0.1% EtOH), which was set to 1. Each condition was performed at least in triplicate. Data were plotted as for Fig. 1. XY plots represent mean + SEM, with **a–c** representative of (**a**) 12, (**b**) 8 and (**c**) 7 independent donor experiments, respectively. Statistical significance was determined by comparing MPA and NET to the vehicle control as well as to each other, using a repeated measures two-way ANOVA with a post hoc Tukey’s multiple comparison test. Symbols *and **denote p < 0.05 and p < 0.01. For comparisons in a, stars denote significance between vehicle vs. MPA, while letters a (p < 0.01) and b (p < 0.0001) denote significance between MPA and NET. In b, stars denote significance between vehicle vs. MPA as well as MPA vs. NET. In c, stars denote significance between MPA vs. NET as well as vehicle vs. NET, while the letter a (p < 0.05) denotes significance between MPA and NET. Detailed statistical analysis is shown in Supplementary Table S4. All data sets except one had all data time points. For (**a**) one of the donor (1) experiments was missing a day 5 value and was therefore excluded from the statistical analysis, although it is included in the final Figure.

Treatment of ectocervical explants in parallel with either vehicle, 100 nM MPA or 100 nM NET showed that MPA significantly increased R5-tropic HIV-1 infection relative to vehicle and NET, while NET had no effect relative to vehicle at any of the time points (Fig. 2a). Similar to results in Fig. 1a, 100 nM MPA significantly increased R5-tropic HIV-1 infection by about 1.2-fold after 3, 7 and 10 days, relative to vehicle (Fig. 2a). Similar results were observed for matched incubations with vehicle, 100 nM MPA or 100 nM NET in endocervical explant tissue, although given the larger error bars in this group of donor experiments and the less robust infection at longer time points, significant effects on HIV-1 replication were only observed for 100 nM MPA at day 5 relative to vehicle (Fig. 2b). Plotting the AUC data showed similar results, with 100 nM MPA significantly increasing R5-tropic HIV-1 infection compared to NET after 10 days, while 100 nM NET showed no significant difference compared to vehicle (see Supplementary Fig. S4). Unlike the results with R5-tropic virus, Fig. 2c shows that 100 nM MPA has no effect on X4-tropic HIV-1 replication over time compared to the matched vehicle control (Fig. 2c), while 100 nM NET significantly decreased X4 HIV-1 replication by about 1.5-fold over time, compared to both the vehicle control at days 7 (1.6-fold) and 10 (1.4-fold), and to MPA at day 5 (1.3-fold), in ectocervical explants (Fig. 2c). Supplementary Table S4 shows the detailed statistical outputs for each data set at each time point. For raw p24 accumulation curves (pg/mL) for select individual donors see Supplementary Fig. S3. Taken together, the data show that 100 nM MPA increases R5-tropic HIV-1 replication in primary ectocervical and endocervical explants, while equimolar NET has no effect, and that the effects of MPA on HIV-1 replication are selective for R5- tropic and not X4-tropic virus.

### MPA increases HIV-1 replication in ectocervical explants via the GR and/or PR

**Figure 3:**
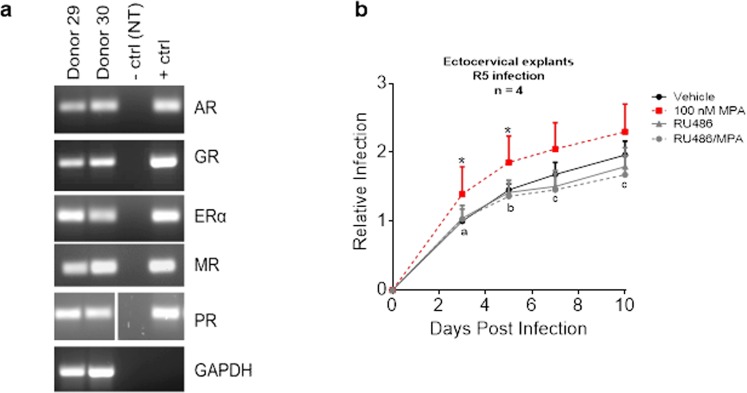
Ectocervical explants express all the steroid receptors and the GR/PR antagonist RU486 significantly reduces the MPA-induced increase in HIV-1_BaL_ replication. (**a**) Ectocervical RNA was screened for steroid receptor and GAPDH mRNA using RT-PCR. Patient donors and controls are labelled on the gel. Positive controls used were expression vectors containing the coding regions of each steroid receptor, respectively. Negative control was the no template control (dH_2_0). Cropped images of stained agarose gels are shown here, while the cropping is explained, and full-length images are shown in Supplementary Fig. S5. (**b**) Ectocervical tissue was incubated for 48 hr in parallel with vehicle, 100 nM MPA, 100 nM RU486, or combinations thereof, followed by R5-tropic HIV-1 infection and media collection at days 3, 5, 7 and 10 post infection. Relative infection was plotted as described for Figs 1 and 2, with the XY plot representative of mean + SEM. Statistical analysis was by repeated measures two-way ANOVA, with a post hoc Tukeys’s test. Statistical significance is denoted by * to indicate p < 0.05 for vehicle vs. MPA, while letters a, b and c represent (p < 0.05), (p < 0.01) and (p < 0.001), respectively, for significance between MPA vs. MPA/RU486. Detailed statistical analysis is shown in Supplementary Table S5. All data sets had all data time points.

Having shown that MPA increases HIV-1 replication in the cervical explants, we sought to determine if the mechanism involves steroid receptor signalling. Initially, we investigated whether steroid receptor mRNAs are expressed in ectocervical tissue. We show for the first time that ectocervical tissue expresses detectable mRNA for all the steroid receptors (Fig. 3a). Thereafter, we used the GR- and PR-selective antagonist RU486 to further investigate the possible involvement of the GR and/or the PR in mediating the observed MPA effect. As observed previously, 100 nM MPA significantly increased R5-tropic HIV-1 replication in the ectocervical explants while RU486 significantly decreased the MPA-induced response. There was no significant difference between the response to vehicle and RU486 alone. For detailed statistical outputs for each data set at each time point see Supplementary Table S5. For raw p24 accumulation curves (pg/mL) for select individual donors see Supplementary Fig. S3. The data suggest that the GR and/or the PR mediate the MPA-induced increased HIV-1 replication in ectocervical explant tissue.

### The mechanism appears to be independent of soluble mediators

**Figure 4:**
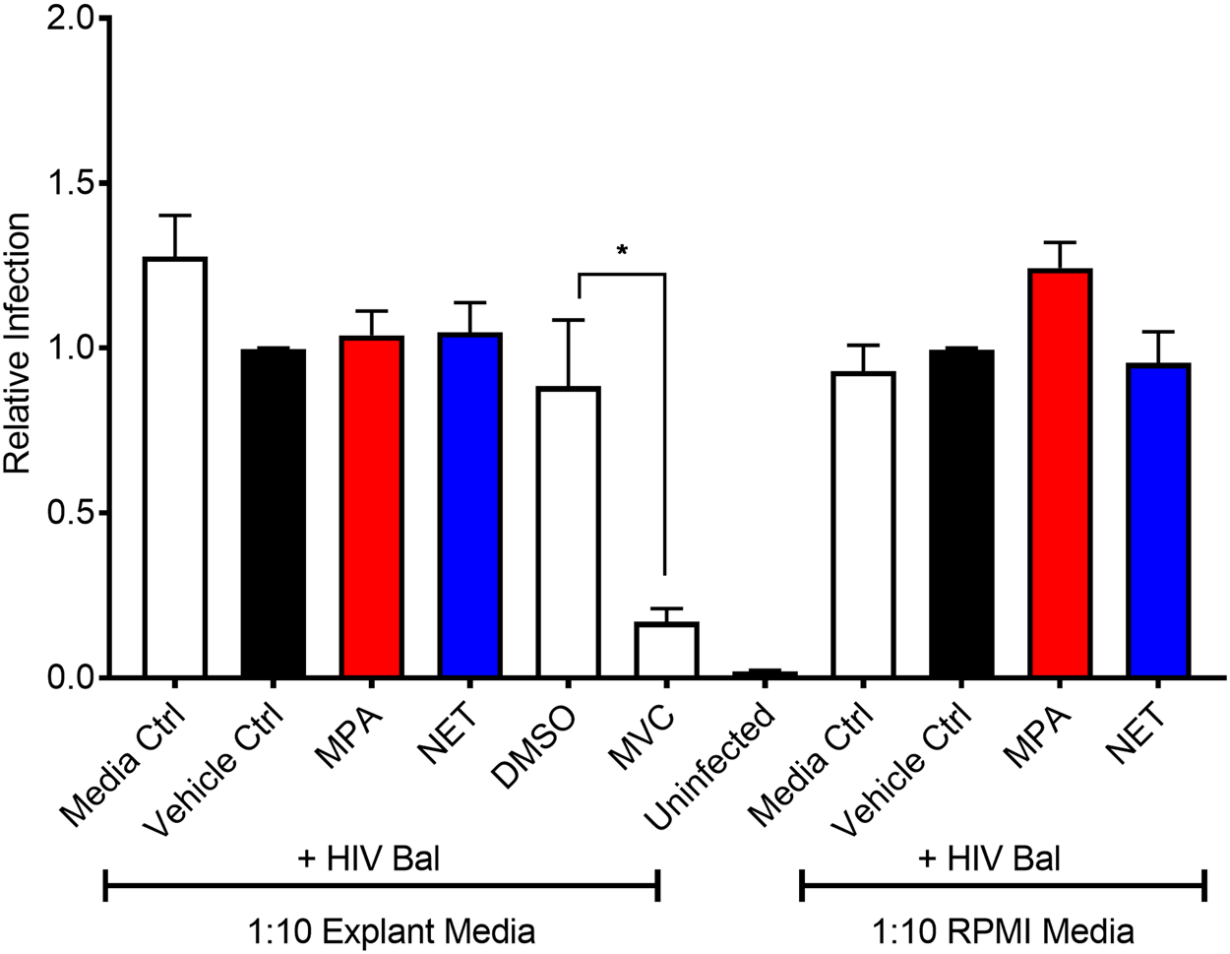
Supernatants of ectocervical explants incubated with MPA or NET have no effect on R5-tropic HIV-1 replication in TZM-bl cells. TZM-bl cells were treated for 24 hr with 1:10 diluted supernatants collected from ectocervical explants previously treated with 100 nM MPA, 100 nM NET, 10 µM Maraviroc (MVC) or with 0.1% EtOH, (v/v) or 0.1% (v/v) DMSO as vehicle controls. Additionally, TZM-bl cells were treated for 24 hr with control RPMI media incubated for 48 hr with 100 nM MPA or NET or vehicle control. Cells were infected with 20 IU of HIV-1_Bal-Renilla_ virus or control virus (uninfected) in the presence of treatments. Cells were harvested 48 hr after infection and infection determined with Brightglo luciferase. Luciferase readings were normalized to cell viability MTT values. The histogram shows the results of nineteen independent explant supernatants, assayed over three independent experiments each performed in duplicate or triplicate and plotted as mean + SEM. Statistical significance was assessed using a non-parametric ANOVA with a post hoc Dunn’s test between conditions with * denoting p < 0.05 between DMSO and MVC.

**Figure 5:**
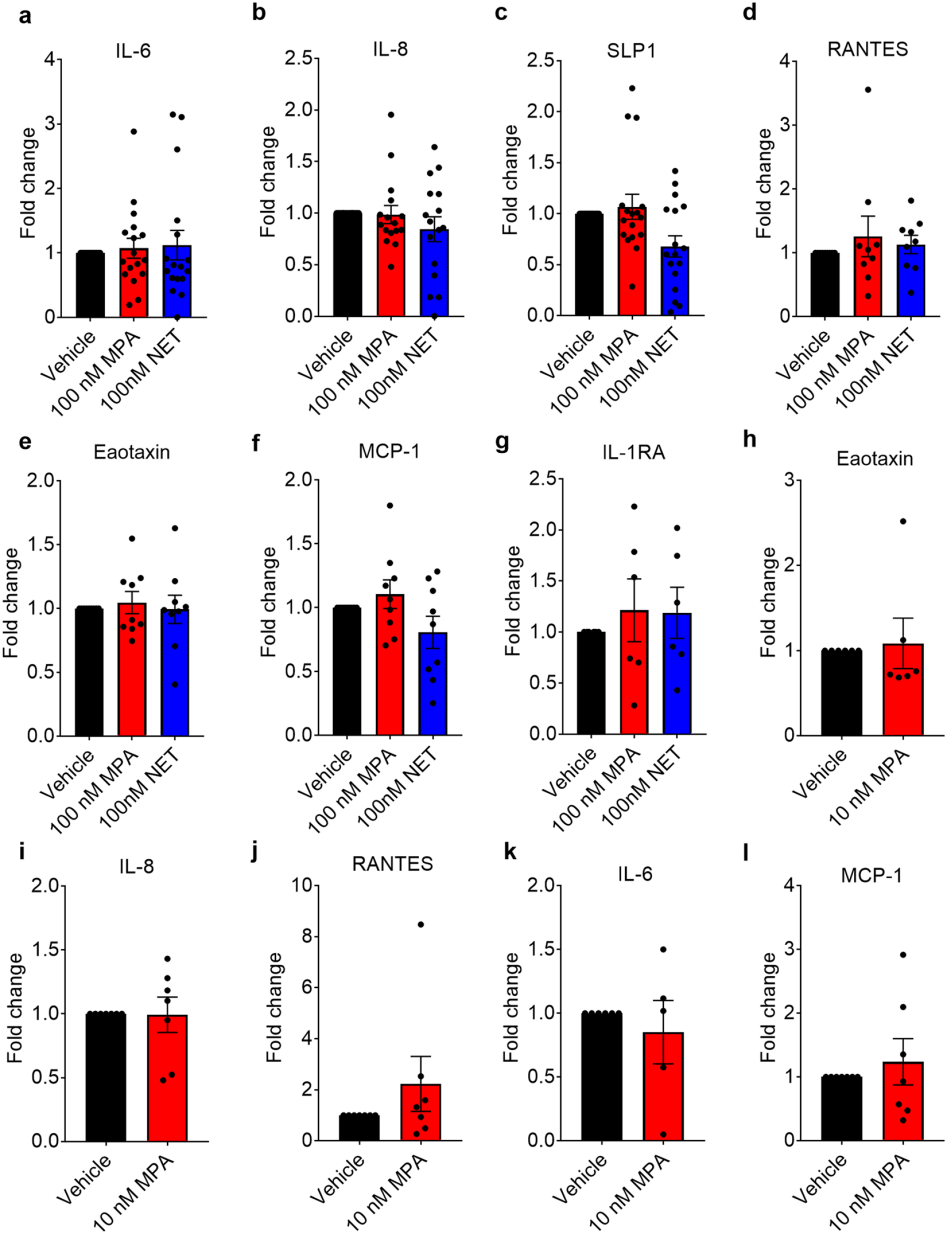
MPA and NET have no significant effects on select secreted protein levels after 48 hr incubation in ectocervical explants. Ectocervical explant tissue samples were stimulated with 100 nM MPA or 100 nM NET (**a**–**g**), or 10 nM MPA (**h**–**l**) and a vehicle control EtOH (0.1% *v/v*) for 48 hr at 37 °C, after which supernatants were collected and stored at −80 °C until use in a DuoSet ELISA, (R&D Systems) (**a**–**c**) or in a Merck Millipore luminex assay (**d**–**l**). In (**a**–**l**) we show histograms of pooled results from five to seventeen donors, plotted as mean ± SEM. Donor numbers varied for each cytokine due to some donor samples falling below detection limits. No significant differences were detected between conditions using non- para metric Kruskal-Wallis one-way ANOVA with a Dunn’s post-test.

We investigated whether changes in soluble mediators induced by 100 nM MPA in cervical explant tissue would affect R5-tropic HIV-1 replication in an add-back experiment using the TZM-bl cervical indicator cell line. The results showed no effect on HIV-1 replication with medium harvested after ectocervical tissue had been incubated for 48 hr with 100 nM of either MPA or NET, as compared to vehicle, suggesting that changes in soluble mediators are not involved (Fig. 4). This result is consistent with our analysis of levels of selected cytokines and chemokines by Luminex and ELISA, which revealed no significant changes in IL6, IL8, RANTES, MCP-1, IL-1RA, SLPI and eotaxin expression levels after treatment with 100 nM MPA or NET for 48 hr, compared to vehicle (Fig. 5 and see Supplementary Table S6).

### MPA, unlike NET, increases CCR5 and CD4 mRNA levels in cervical tissue

**Figure 6:**
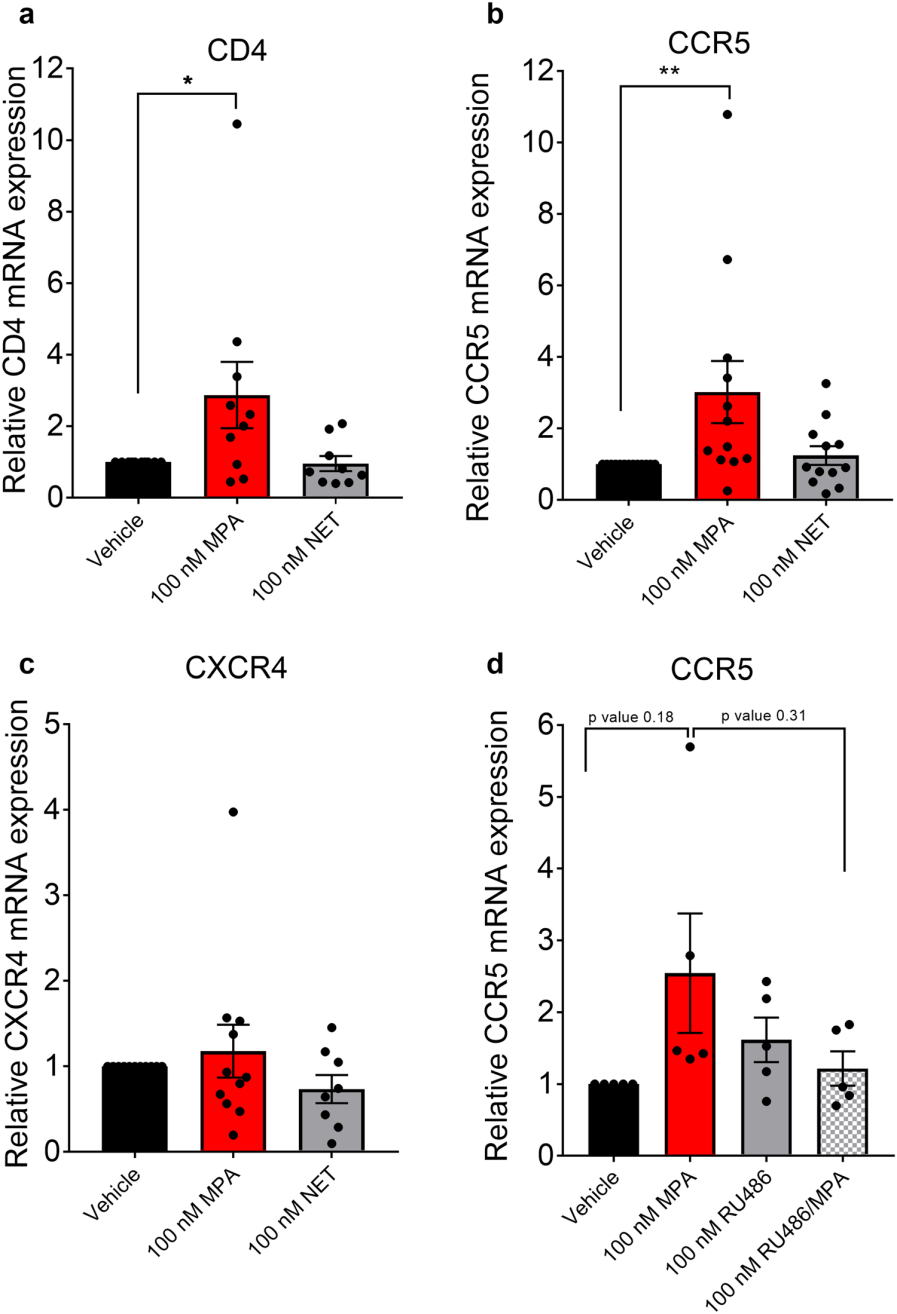
MPA, unlike NET, increases CCR5 mRNA levels in cervical explant tissue in a GR/PR-dependent manner. (**a**–**c**) Ectocervical explant tissue were stimulated with 100 nM MPA, 100 nM NET or vehicle 48 hr or (**d**) treated for 48 hr with vehicle control (ETOH) (0.1% v/v ethanol), 100 nM MPA, 100 nM RU486 (RU), or combinations thereof (RU/MPA). RNA was isolated, cDNA was synthesised and relative CCR5 mRNA levels were determined by real time qPCR, normalised to GAPDH. Relative fold change in expression was determined by setting vehicle control to 1. (**a**) Shows the results for CD4 from 10 (MPA) and 9 (NET), while (**b**) shows results for CCR5 from 12 (MPA and NET) and (**c**) shows results for CXCR4 from 11 (MPA) and 8 (NET), independent donor experiments, respectively. (**d**) The histogram shows pooled results from five independent experiments with vehicle set to one, plotted as mean ± SEM. Statistical significance was assessed by a non-parametric one-way ANOVA Kruskal-Wallis with post hoc Dunn’s tests between conditions (**d**) or a non-parametric Wilcoxon Signed Rank test with the median set to 1 (**a**–**c**).

Having shown that MPA but not NET increases R5-tropic HIV-1 replication in explant tissue, we next investigated whether these responses were due to differential effects on CD4 or HIV-1 co-receptor mRNA expression levels. We found that incubation of ectocervical tissue explants for 48 hr with 100 nM MPA significantly increased CD4 mRNA levels by 2.6-fold ±0.87 SEM (p = 0.042), while 100 nM NET had no effect (Fig. 6a). Moreover, 100 nM MPA significantly increased expression of CCR5 mRNA by 2.8-fold ±0.82-SEM (p = 0.0215), while equimolar NET had no effects (Fig. 6b). Neither 100 nM MPA nor 100 nM NET had any significant effect on CXCR4 mRNA levels (Fig. 6c). The upregulation of CCR5 expression by MPA (2.54 fold ±0.8321 SEM) was inhibited by co-incubation with RU486 (1.216 fold ±0.2386 SEM) in the ectocervical explant tissue, supporting a role for the GR and/or PR in this response (Fig. 6d) as found for MPA-induced HIV-1 replication.

### MPA, unlike NET, increases the relative frequency of CD4+ cells and activated monocytes in cervical tissue

**Figure 7:**
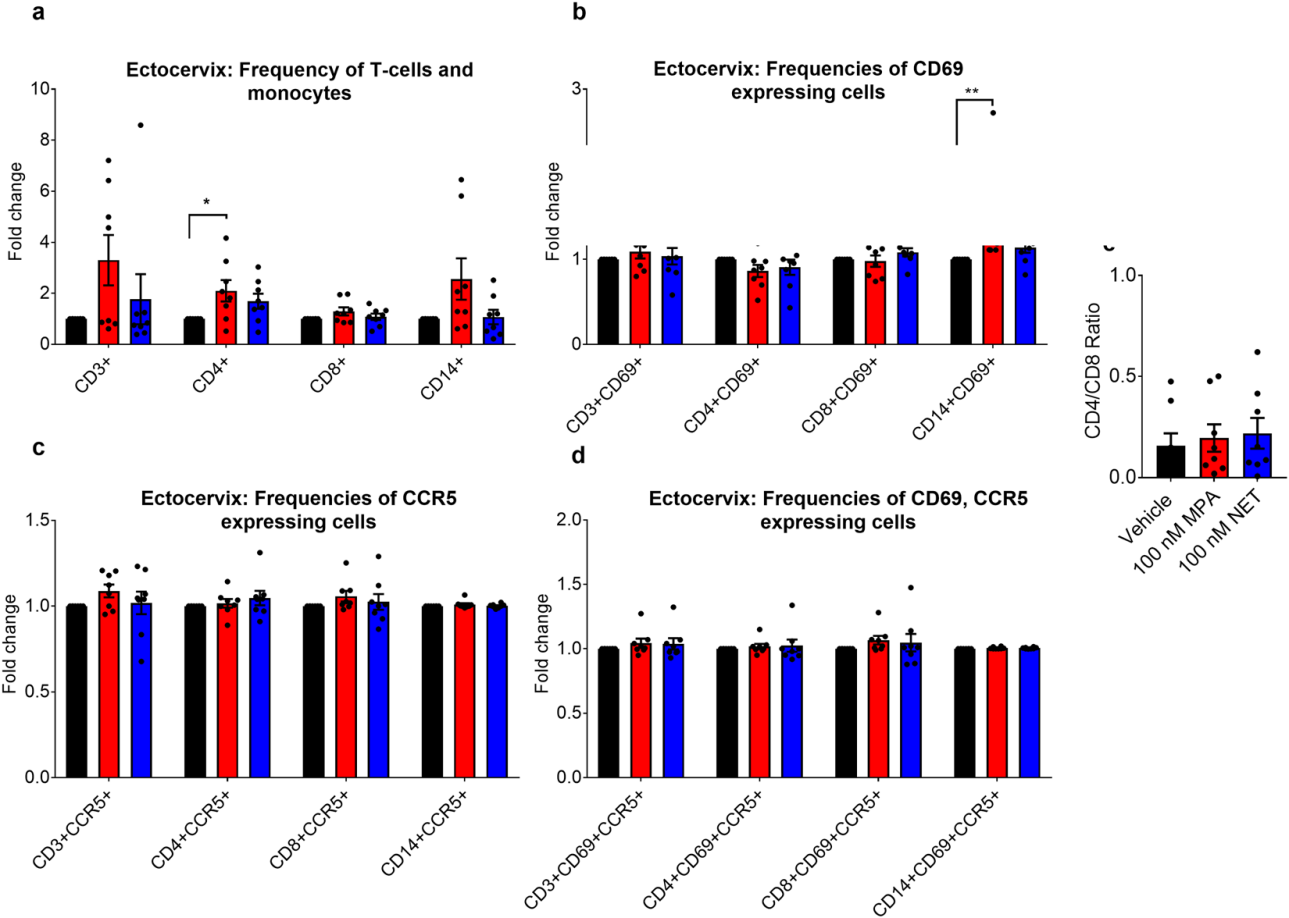
MPA significantly increases relative frequency of CD4+ cells and CD14+ CD69+ expressing cells after 7 days. Ectocervical explant tissue were stimulated in parallel with vehicle (EtOH) or 100 nM MPA or 100 nM NET for 7 days after which the relative levels of (**a**) CD3+, CD4+ and CD8+ and CD14 T cells (**b**) CD69 and (**c**) CCR5 or (**d**) CD69, CCR5 expressing CD3+, CD4+ and CD8+ and CD14 T cells were determined using flow cytometry. Results show the fold changes of frequency of 8 independent experiments, where the % cells for vehicle for each donor sample was set to 1 and the relative frequency for matched MPA and NET incubations was calculated relative to that donor vehicle value. (**e**) CD4/CD8 ratios of the 8 independent experiments were determined. Histograms are representative of mean ± SEM. Statistical significance was determined by using a non-parametric Kruskal-Wallis one-way ANOVA with a Dunn’s post-test with * or ** denoting p < 0.05 or p < 0.01, respectively.

Towards understanding the differential effects of MPA and NET on HIV-1 replication, we investigated whether 100 nM MPA or NET affected the frequency, activation or CCR5 levels of CD3+ and CD14+ cells in ectocervical tissue by flow cytometry after 48 hr or 7 days. For the gating strategy see Supplementary Fig. S6. Most of the cells analysed after treatment for 48 hr or 7 days were viable, although there was evidence for some cell death in about 10% of samples (see Supplementary Fig. S7 and Supplementary Table S7). Neither MPA nor NET appeared to have any significant effects on frequency or activation (as assessed by CD69) of CD4+, CD8+ or CD14+ cells, or their CCR5 expression levels, after 48 hr (see Supplementary Table S8). Interestingly, incubation for 7 days revealed that 100 nM MPA significantly increased the relative frequency of CD4+ expressing T cells and activated CD69+ CD14+ cells, unlike 100 nM NET, relative to vehicle control for each matched ectocervical donor tissue (Fig. 7 and see Supplementary Table S9A). No significant differences were detected between the effects of MPA and NET on the relative frequency of CD69+ or total CD8+ or CD14+ cells (Fig. 7 and see Supplementary Table S9A). Representative scatter plots showing an increased frequency of CD4+ cells, but not CD8+ cells are given in Supplementary Fig. S8. Furthermore, MPA and NET had no effect on the relative frequency of CCR5-expressing cells or the density (as measured by median florescent intensity (MFI)) of CCR5 expression (Fig. 7 and see Supplementary Table S9A and B) after 7 days. The total frequencies of CD3, CD4, CD8 and CD14 were lower after 7 days compared to 48 hr, with the most dramatic decrease seen for CD4+ cells (see Supplementary Tables S8A and S9A). Concomitantly, the CD4/CD8 ratio was also 4-fold lower after 7 days compared to 48 hr. For the absolute numbers of CD4+ and CD4+ CCR5+ cells for individual donors see Supplementary Table S10. No significant change in CD4 density (MFI) was observed between treatment groups (see Supplementary Table S11).

## Discussion

We show for the first time that physiologically relevant concentrations of MPA have direct effects on cervical explant tissue *ex vivo*, to significantly increase R5-tropic, but not X4-tropic HIV-1 replication. The effects of MPA on R5-tropic viral replication in endocervical tissue are likely to be physiologically relevant, since 10 nM MPA falls well within the range of average reported peak serum levels of DMPA-IM users, with the Cmax after 8 injections with DMPA-IM being reportedly about 11 nM^55^. However, it should be noted that the concentration of MPA and NET in genital tract tissue is unknown and may or may not differ substantially from that measured in serum. Since 1 nM MPA had no significant effects on HIV-1 replication, it is possible that the lower dose DMPA-SC may have less of an effect on HIV-1 replication than DMPA-IM in FGT cervical tissue *in vivo*, although the effects *ex vivo* may not be translatable *in vivo*. Our findings showing no significant increase with 100 nM NET suggest that NET-EN is unlikely to affect HIV-1 replication even at peak serum NET concentrations via any mechanism that is conserved in our *ex vivo* model. Interestingly we observed similar results and MPA dose-dependency for HIV-1 replication in PBMCs^65^ suggesting similar types of HIV-1 target cells and MPA-increased infection mechanisms are involved in the genital tract as in PBMCs. Bearing in mind the limitations of extrapolating *ex vivo* to *in vivo* data, our current results are consistent with the limited clinical epidemiological data showing a significant increased risk of HIV-1 infection for DMPA-IM compared to NET-EN users. It would be ideal to perform these studies with transmission-founder clinical isolates, but other reports have shown that these do not readily replicate detectably in *ex vivo* tissue models^82^. Our findings further suggest that at least some effects of MPA *in vivo* may occur via direct effects on FGT endocervical and ectocervical tissue to increase HIV-1 replication, unlike for NET.

HIV-1 acquisition in the FGT is likely to involve multiple mechanisms, including effects on HIV-1 replication^12^. Several *in vivo* mechanisms would not be detected in our non-polarized *ex vivo* model, such as changes in FGT barrier integrity and permeability. Indeed data from mouse and clinical models show that MPA increases the permeability of the genital barrier^15^ and increases HIV-1 transcytosis in primary genital epithelial cells^31^. The *ex vivo* explant model also has some other limitations^83^, but several biological repeats and using fresh tissue still enable meaningful significant results^84,85^. Nevertheless, the advantages of the *ex vivo* model are that direct effects on the FGT relevant to HIV- 1 acquisition and replication using the same known concentrations of specific progestins compared to vehicle can be investigated in parallel in the same donor sample, unlike for clinical studies, which also involve indirect effects and multiple confounding behavioural factors such as condom usage.

Other mechanisms whereby contraceptives may affect HIV-1 acquisition in the FGT include changes in soluble mediators of inflammation and innate immunity^12^. Several clinical studies show a correlation between increased FGT inflammation and increased HIV-1 acquisition^86,87^. However, the reported effects of contraceptives in the FGT on these mediators, including IL-6, IL-8 and RANTES, are inconsistent^13,16–19,88–90^. Expression of these and other select mediators are regulated directly by MPA in genital tract primary or cell line models *ex vivo*, both in a pro- and an anti-inflammatory manner, at physiologically relevant concentrations, but not by NET, where investigated^31,32,41,91,92^. Whether MPA or NET have direct effects on expression of such soluble mediators in cervical tissue is unknown. The results of our add-back experiments in the TZM-bl indicator cell line suggest that MPA increases R5 HIV-1 replication without a requirement for detectable changes in levels of select soluble secreted mediators from the explant tissue. This is supported by our lack of detection of any significant changes in levels of select soluble mediators in explant media in response to both 10 or 100 nM MPA or 100 nM NET. Nevertheless, our findings do not exclude a role for changes in other soluble mediators not investigated, nor the possibility that significant small effects undetectable by the power of our study do occur with the mediators investigated, or that longer exposure to progestins may reveal significant changes.

Another possible mechanism whereby contraceptives may increase HIV-1 acquisition in the FGT is via increased frequency of CD4+ CCR5+ T cells or density of CCR5 expression on CD4+ T cells^12^. This is consistent with our data showing that MPA increased R5-tropic and not X4-tropic viral replication in ectocervical explant tissue. CCR5 is reported to be the predominant co-receptor for mucosal HIV-1 infection in humans and is expressed on T cells, dendritic cells and macrophages in the mucosal tissue of the FGT^74,93^, while CCR5+ CD4+ cells are the primary HIV-1 targets for infection^94–97^, consistent with results from another *ex vivo* cervico-vaginal tissue model showing predominant R5-tropic infection^98^. Our novel finding that MPA but not NET increases total cervical tissue levels of CCR5 but not CXCR4 mRNA suggests that a likely mechanism whereby MPA but not NET increases HIV-1 replication in the explant model is via upregulation of CCR5 expression levels on HIV-1 target cells. This is consistent with data showing that cytobrushes from women on injectable contraceptives (76% on DMPA-IM) contain a greater frequency of CCR5+ CD4+ cells and increased density of CCR5 on CD4+ cells, compared to no contraception^13^. Similarly, the frequency of CCR5 expressing cells is significantly increased in vaginal biopsy tissue of women on DMPA-IM, compared to no contraception^99^. Others, however, did not detect any change in CCR5 expression levels on T cells in cervicovaginal lavages (CVL)^17^, or mucosa cells isolated from cytobrush samples from women on DMPA-IM^88^, possibly due to differences in the intrinsic factors of sample populations, methodology or differences in cells between lavage, cytobrushes and tissue. Interestingly, frequency of CCR5+ CD4+ T cells was shown to be higher in DMPA-IM-treated macaques than in an E2-treated group in vaginal tissue^100^, suggesting a similar mechanism in macaques. However, our flow cytometry analysis of collagenase digested tissue did not reveal any changes in density of CCR5 expression (MFI) or the frequency of expression of CCR5 on CD3+ or CD14+ cells, after 48 hr or 7 days exposure to 100 nM MPA. Furthermore, flow cytometry showed that after 48 hr, 96% of CD4+ and 99% of CD8+ cells and after 7 days 88% of CD4+ and 88% of CD8+ cells, already expressed CCR5, independent of treatment, making it unlikely that a further increase could be detected or relevant.

Our CD14+ findings at 48 hr are consistent with previous reports showing CD14+ cells to be the predominant cell type in ectocervical tissue^80,101^. The overall decrease in different cell type frequencies between 48 hr and 7 days may be indicative of a general depletion of CD3+ and CD14+ cells over the 7 days in culture, in particular the CD4+ cells, or an overall increase in other cell types. The higher CD4/CD8 ratio for vehicle-treated tissue at 48 hr compared to 7 days was similar to a previously reported CD4/CD8 ratio in ectocervical explant tissue^80^. However, comparisons between our 48 hr and 7 day data should be interpreted with caution since the 48 hr donors were not matched with the 7 day donors and there is a high degree of inter-donor variability at both time points. The reported high percentages of CCR5 and CD69 expressing cells in our study are consistent with findings by others that the majority of explant CD4+ cells express CCR5 or CD69 following three days in culture^102^, as well as findings from freshly harvested cervico-vaginal tissue explants where the majority of CD4+ and CD14+ cells express CCR5^103^. In contrast, others report lower percentages for CCR5 expressing CD4+ cells (28%) and CD8+ cells (16%) within a CD3+ population of T cells made up of 41% CD4+ or 59% CD8+ from freshly harvested and digested tissue^80^ or CD4+ cells harvested for cervical lavages (CVL)^104^. Differences in cell populations between studies may indicate that cells have undergone different degrees of change in surface marker expression, general immune activation, induction of hypoxia or apoptosis^82,105^, or induction of wound repair upon mechanical stress^105^ due to the harvesting procedures used to generate single cells for flow cytometry analysis and may not accurately reflect prior cell populations. Alternatively, differences may be due to intrinsic donor population differences. Disparity in digestion protocols, with different collagenases exerting differential effects on CCR5 expression levels^106^ or a variety of sampling sources of cervical samples (cytobrushes, lavages), which are shown to have varied levels of CD4+ cells^80,104,107,108^, may also account for such differences. We do show that the cells subjected to flow cytometry analysis after 7 days of exposure to 100 nM MPA remain viable. General immune activation and increased CCR5 expression induced by flow cytometry sample preparation could have masked an MPA-induced increase in CCR5 expression. In support of this possibility, we have recently shown that PBMCs investigated by flow cytometry without the collagenase procedure have lower basal CCR5 expression and exhibited an increased CCR5 density (MFI) and frequency of CCR5 expressing CD4+ (21.03% ± 4.466), CD8+ and CD14+ cells after incubation with MPA for 7 days^65^. However, this could also reflect real differences in the PBMC versus tissue immune responses to MPA^13^. Immunohistochemical staining on whole tissue slices may be a better way to decipher the cell type (s) origin of the increased CCR5 mRNA shown in our study, which could still possibly be from CD4+, CD8+ or CD14+ cells as found for PBMCs, or alternatively, involve other cell types not investigated by flow cytometry, such as epithelial cells or stromal fibroblasts also present in cervical tissue and expressing CCR5^109^. Future experiments using markers for these different cell types could be used to address these questions but are beyond the scope of the present study.

The increase in the relative frequency of CD4+ cells after 7 days coupled to the lack of possible recruitment of additional cells in an explant model suggests that the upregulation of the CD4 receptors on CD3+ cells not previously expressing CD4 could be a possible explanation for the increased HIV replication observed with MPA, since CD4 is also required for HIV-1 infection. Consistent with this possibility is that after 48 hr we detected an increase in CD4 mRNA. Alternatively, one could speculate that MPA partially prevents down-regulation of CD4 surface markers on CD3+ cells, compared to vehicle and NET. Other studies did not detect an increase in the frequency of CD4+ cells by immunohistochemical staining of vaginal biopsies from women on DMPA-IM compared to no hormone controls^110^, possibly due to differences in methodology or target tissue. Our explant data are consistent with our findings in PBMCs where we also see an increased relative frequency of CD4+ CD3+ T cells and an increased expression of both CD4 and CCR5 but not CXCR4 mRNA in TZM-bl cells with MPA^65^. Interestingly, a recent clinical study also supports a role for increased CCR5 expression and activation of systemic CD4+ T cells in increasing infectability of CCR5+ CD4+ T cells in PBMCs from women on DMPA-IM^111^. However, it should be noted that FGT and systemic HIV-1 target cells are different, although some similarities exist^13,112^. While the role of CD69+ CD14+ monocytes in HIV-1 infection has not been elucidated, several studies show that changes in activation of CD14+ cells such as macrophages, which are permissible to R5 HIV-1 infection^113^, are reported to change their susceptibility to HIV-1 infection^114–116^. Our results showing increased activation of macrophages in the cervical explants by MPA after 7 days are consistent with activated macrophages playing a role in driving increased HIV-1 replication in CD4+ CCR5+ T cells in cervical tissue. The finding that removal of CD14+ cells from PBMCs inhibits the effect of MPA on increased HIV-1 replication^30^ also appears to be consistent with our study, suggesting that monocytes play a role in increased infection of T cells by MPA. Alternatively, or in addition there may be an increase in HIV-1 replication in monocytes^113^.

Our data collectively suggest that the mechanism via which MPA, unlike NET, increases HIV-1 replication in cervical tissue is mediated via the GR. Although we show that cervical tissue express GR, PR and AR, it has been previously established that at 100 nM MPA, only the GR is likely to discriminate between MPA and NET^32,34,36,117,118^. MPA and NET exert differential effects on expression of select genes at 100 nM via the GR in several model systems^32,36–38^. Additionally, MPA and NET are both PR agonists with affinities in the nanomolar range^34,118^ and thus would be expected to elicit similar responses at 100 nM, if via the PR. The PR that we detected in the cervical explant tissue most likely originates from fibroblast cells^119^, and not from CD3+ or CD14+ cell types or primary epithelial cells^32,76,119^. Since stromal fibroblasts are reported to enhance HIV-1 infection in susceptible CD4+ T cells^120^, it is also possible that the PR plays a role in HIV-1 replication in our explant model, although unlikely in contributing to the differential actions of MPA and NET. In support of a role for the GR in explant tissue, we observed similar differential effects with MPA and NET on HIV-1 replication in PBMCs and TZM-bl cells, which do not express PR^65^, as in the current study. Using a more specific GR or PR antagonist would have been helpful to discriminate between GR- and PR-mediated effects. However, some such reported antagonists^121^ are not commercially available. We did investigate the use of one non-commercial and reportedly GR-specific antagonist^122^, but this had a very low affinity for the GR in our hands^36^, such that no antagonism would have been observed even at 1 µM if the GR was involved and its use was thus not suitable for these experiments. Further experiments would be required to definitively establish the roles of the GR and PR in these responses in tissue.

In conclusion, our data support a mechanism whereby MPA, at concentrations found in serum of DMPA-IM users, unlike NET, directly increases R5 but not X4 HIV-1 replication in CD4+ T cells via increasing expression of CD4 and CCR5 co-receptors on T cells and activation of monocytes, to increase HIV-1 infection in cervical tissue. The detected differences in the actions of MPA compared to NET are likely to be GR-mediated. Our findings suggest that MPA could increase HIV-1 acquisition by the above mechanism once the virus has crossed the epithelial barrier, in the absence of factors such as hypoestrogenism and changes in the microbiome and soluble mediators. However, our data do not exclude a contributing role for any of these or other mechanisms. Furthermore, our data do not exclude the possibility that the MPA but not NET responses require the presence of other signalling molecules, such as IL-2, present in the media. Indeed, it is likely that MPA, acting alone or together with other signalling pathways, exerts effects on multiple genes and physiological processes relevant to several mechanisms involved in HIV-1 acquisition, given the ubiquitous expression of the GR. While it is unclear and difficult to establish what the impact of the MPA-induced increased HIV-1 replication in the *ex vivo* model would be on HIV-1 acquisition *in vivo*, this study does suggest at least one mechanism whereby the impact may be different for MPA compared to NET. Our study adds to the body of evidence showing that MPA and NET act differently regarding several biological mechanisms relevant to HIV-1 acquisition and supports disaggregation of DMPA-IM and NET-EN regarding HIV-1 acquisition risk.

## Supplementary information

Supplementary Tables and Figures

## Data Availability

The datasets generated during and/or analysed during the current study are available from the corresponding author on reasonable request.
