# Supplementary material for: The contraceptive medroxyprogesterone acetate, unlike norethisterone, directly increases R5 HIV-1 infection in human cervical explant tissue at physiologically relevant concentrations
Source: Sci Rep. 2019 Mar 13;9:4334. doi: 10.1038/s41598-019-40756-7 (PMC6416361; doi:10.1038/s41598-019-40756-7)
Supplement: Supplementary file 1 — Supplementary Tables and Figures [file 41598_2019_40756_MOESM1_ESM.pdf]

Supplementary Tables and Figures

The contraceptive medroxyprogesterone acetate, unlike norethisterone, directly increases R5 HIV-1 infection in human cervical explant tissue at physiologically relevant concentrations

Roslyn M. Ray[#,1], Michelle F. Maritz[#,1], Chanel Avenant[#1], Michele Tomasicchio[2], Sigcinile Dlamini[1], Zephne van der Spuy[3] and Janet P. Hapgood[1,4, *]

Supplementary Tables

Supplementary Table S1: Hormone value ranges for different phases of the menstrual cycle taken from NHLS.

	Follicle stimulating hormone(IU/L)	Luteinising hormone (IU/L)	Oestradiol (E2) (pmol/L)	Progesterone (nmol/L)
**Follicular phase:**	3.5 - 12.5	2.4 - 12.6	45 – 854	0.2 - 2.8
**Ovulation phase:**	4.7 - 21.5	14.0 - 95.6	151 - 1461	0.4 - 38.1
**Luteal phase:**	1.7 - 7.7	1.0 - 11.4	82 – 1251	5.8 - 75.9
**Postmenopausal:**	25.8 - 134.8	7.7 - 58.5	<18 – 505	<0.2 - 0.4

Supplementary Table S2: Donor Table

Donor	HSV 1&2 IgG	HSV 1 IgG	HSV 2 IgG	HSV IgM	Hormone levels LH (IU/L)	FSH (IU/L)	E2 (pmol/L)	P4 (pmol/L)	Age	Stage of menstrual cycle	Indication for surgery	Figure used
1	positive				5.5	8.7	83.7	9.1	51	Follicular	Ultrasound showed hemipelvic mass	Figs. 1a, 2a
2		positive	positive	negative	10.4	6.1	230.4	1.6	44	Follicular	Abnormal uterine bleeding (Menorrhagia), failed medical treatment, Anaemia	Figs. 1a, 2a, 2c
3		positive	negative	negative	9	6	2629	0.9	48	Irregular prolonged bleeding	Fibroid uterus, Menorrhagia, prolonged menses, Dysmenorrhea	Figs. 1a, 2a
4		positive	negative	negative	3	5.9	84.2	0.5	44	Follicular	Fibroid uterus, Menorrhagia, Anaemia	Figs. 1a, 2a, 2c
5	positive				3.2	4.1	462.6	40.5	53	Perimenopausal - Luteul	Ovarian mass (likely benign)	Figs. 1a, 1d, 2a, 2b, 5a-h
6	positive				6.9	14.7	395.2	16.5	41	Early luteal	Unknown	Figs. 1a, 2a, 5a-h, 6
7	positive				Nd	Nd	Nd	Nd	42	Irregular	Multi-fibroid uterus	Figs. 1a, 1b, 1d, 2a, 2b, 5a-h
8	positive				1.6	4.3	238	7.9	46	Luteal	Multi-fibroid uterus	Figs. 1a, 1d, 2a, 2b, 3c, 5a-h
9	positive				0.9	3.2	128	6.6	47	Luteal	Unknown	Figs. 1a, 1d, 2a, 2b, 5a-h
10		positive	negative	negative	11	11.3	179	2.4	49	Follicular	Abdominal distension, abdominal pain	Figs. 1a, 1b, 1c, 2a, 5a-h, 5i-l
11		positive	negative	positive	13.1	10.5	231	8.5	48	Ovulatory	Urinary incontinence, uterine prolapse, Grade II cystocele, Grade I uterine prolapse & rectocele	Figs. 1a, 1b, 1e, 5a-l
12	positive				21.6	43.2	<19	<0.7	53	Perimenopausal - Irregular	Multi-fibroid uterus	Figs. 1a, 1b, 1c, 1d, 2b

Donor	HSV 1&2 IgG	HSV 1 IgG	HSV 2 IgG	HSV IgM	Hormone levels LH (IU/L)	FSH (IU/L)	E2 (pmol/L)	P4 (pmol/L)	Age	Stage of menstrual cycle	Indication for surgery	Figure used
13		positive	negative	positive	7.2	7.7	286.4	0.3	49	Follicular Phase	Adenomyomatous uterus, Nabothian cyst on cervix	Figs. 1a, 1b, 1c, 5i-l
14		positive	negative	negative	5.1	2.8	390	7.2	45	Luteal phase	Pelvic organ prolapse	Figs. 1a, 1b, 1c, 1d, 2b, 5i-l
15		positive	negative	positive	7.8	6.4	329	19.1	46	Luteal Phase	Pelvic organ prolapse III procedentia	Figs. 1a, 1b, 1c, 5i-l
16	Nd				29.6	61.3	120	2.9	46	Perimenopausal - Irregular	Fibroid uterus	Figs. 1a, 1b, 1e, 3c, 5a-h, 5i-l
17	positive				1.3	5.2	33	<0.7	47	Perimenopausal - Irregular	Uterine prolapse	Figs. 1a, 3c, 5a-h
18	positive				0.6	3.7	?	2.6	46	Perimenopausal - Irregular	Multi-fibroid uterus	Figs. 1a, 3c
19	Nd				3.8	6.3	270	6.6	Nd	Luteal		Fig. 1a
20	positive				0.8	4.1	52	Nd	42	Irregular	Unknown	Fig. 5a-h
21	positive				Nd	Nd	Nd	Nd	44	Irregular	Uterine polyp	Fig. 1b
22	positive				4.3	3.9	19	2.9	54	Follicular	Fibroid uterus, history of dysmenorrhea & menorrhagia	Figs. 1e, 5i-l
23	positive				Nd	Nd	Nd	Nd	45	Irregular prolonged bleeding	Multi-fibroid uterus	Fig. 1e
24		positive	positive	negative	12.1	23.2	46	<0.1	39	Irregular	Heavy menstrual bleeding	Fig. 1e
25		positive	negative	negative	4.4	4.8	163.5	1	37	Follicular	Fibroid uterus, heavy menstrual bleeding	Fig. 2c
26		positive	negative	negative	35.7	63.8	39.3	0.5	46	Perimenopausal - Irregular	Uterine prolapse	Fig. 2c

Donor	HSV 1&2 IgG	HSV 1 IgG	HSV 2 IgG	HSV IgM	Hormone levels LH (IU/L)	FSH (IU/L)	E2 (pmol/L)	P4 (pmol/L)	Age	Stage of menstrual cycle	Indication for surgery	Figure used
27		negative	negative	negative	5.6	6.3	476.3	0.9	38	Follicular	Menorrhagia for two years	Fig. 2c
28		positive	positive	negative	0.6	3.3	212.4	0.5	41	Late Luteal	Abnormal uterine bleeding, single fibroid	Fig. 2c
29	Nd				Nd	Nd	Nd	Nd	49	Irregular	Multi-fibroid uterus	Figs. 3a, 6
30	positive				1.6	3.7	107	1.1	52	Perimenopausal	Endometrial Hyperplasia	Figs. 3a, 6
31	positive				Nd	Nd	Nd	Nd	44	Irregular	Multi-fibroid uterus	Fig. 4
32	positive				Nd	Nd	Nd	Nd	44	Irregular	Multi-fibroid uterus	Fig. 4
33	positive				Nd	Nd	Nd	Nd	43	Irregular	Multi-fibroid uterus, symptomatic anaemia	Fig. 4
34		positive	negative	negative	4.4	8.8	313	<0.1	48	Irregular prolonged bleeding	Menorrhagia - fibroid uterus	Figs. 4, 5a-h, 48h FACS
35		positive	positive	negative	1.9	4.6	95	0.3	50	Follicular	Heavy menstrual bleeding due to multi-fibroid uterus	Figs. 4, 5a-h, 48h FACS
36					<0.1	0.1	<19	0.6	40	Follicular	Abnormal uterine bleeding, failed medical management	Figs. 4, 5a-h
37		positive	negative	negative	12	14	74	1.4	43	Irregular	Multi-fibroid uterus	Figs. 4, 5a-h
38		positive	negative	negative	22.6	12.2	922	0.3	44	Follicular	Multi-fibroid uterus	Figs. 4, 5a-h, 48h FACS
39		positive	negative	negative	1.2	0.8	227	<0.2	40	Irregular	Vesicovaginal fistula repair	Figs. 4, 5a-h
40		positive	negative	negative	<0.1	0.1	<19	0.9	44	Irregular	Multi-fibroid uterus, abnormal uterine bleeding	Figs. 4, 5a-h, 7

Donor	HSV 1&2 IgG	HSV 1 IgG	HSV 2 IgG	HSV IgM	Hormone levels LH (IU/L)	FSH (IU/L)	E2 (pmol/L)	P4 (pmol/L)	Age	Stage of menstrual cycle	Indication for surgery	Figure used
41		positive	negative	negative	0.7	5.3	27	2.8	45	Follicular	Multi-fibroid uterus	Figs. 4, 5a-h, 7
42		positive	negative	negative	6.8	7.9	163	<0.2	45	Follicular	Abnormal uterine bleeding	Figs. 4, 5a-h, 7
43		positive	positive	negative	5.5	12.2	131	2	49	Follicular	Multi-fibroid uterus	Figs. 4, 5a-h, 7
44		positive	negative	negative	40.9	49	349	0.2	51	Perimenopausal - Irregular	Uterine fibroid	Figs. 4, 5a-h, 7
45		positive	positive	negative	14.3	11.1	313	8.2	51	Perimenopausal - Irregular	Abnormal uterine bleeding	Figs. 4, 5a-h, 7
46		positive	negative	negative	2.1	1.1	30	<0.1	51	Irregular prolonged bleeding	Heavy prolonged menstrual bleeding due to multi-fibroid uterus	Figs. 4, 5a-h, 7
47		Positive	negative	negative	12.5	17.9	196	3.6	40	Irregular prolonged bleeding	Abnormal uterine bleeding, Ovarian cyst	Figs. 4, 5a-h, 7
48		Positive	negative	negative	37.9	63.9	60	<0.1	53	Irregular prolonged bleeding	Fibroid uterus, pressure symptoms, abnormal uterine bleeding, failed medical treatment	Figs. 4, 5a-h
49		Positive	positive	negative	2.8	4.2	164	4	43	Luteul	Heavy menstrual bleeding	Figs. 4, 5a-h
50	positive				4.6	4	162	Nd	51	Irregular prolonged bleeding	Abnormal uterine bleeding	Figs. 5a-h, 6
51	positive				1.4	3.6	Nd	7.6	41	Luteal	Multi-fibroid uterus	Figs. 5a-h, 6
52	positive				9.3	17.5	99.9	< 0.7	46	Follicular	Abnormal uterine bleeding with multi-fibroid uterus	Fig. 6
53	Nd				1.1	3.2	<19	2.6	42	Irregular	Fibroids causing urinary symptoms	Fig. 6

Donor	HSV 1&2 IgG	HSV 1 IgG	HSV 2 IgG	HSV IgM	Hormone levels				Age	Stage of menstrual cycle	Indication for surgery	Figure used
					LH (IU/L)	FSH (IU/L)	E2 (pmol/L)	P4 (pmol/L)				
54	positive				4.3	8.9	100	0.8	36	Irregular prolonged bleeding	Abnormal uterine bleeding	Fig. 6
55	positive				0.5	2.4	132	5.7	34	Irregular	Multi-fibroid uterus	Fig. 6
56		Positive	negative	negative	7.2	6.9	413	0.6	44	Irregular	Fibroid uterus, heavy menses, pelvic pain	Fig. 6
57		Positive	negative	negative	6.3	6.1	537.1	0.3	48	Follicular	Fibroid uterus, backache, urinary frequency, abnormal uterine bleeding	Fig. 6
58		Positive	positive	negative	4.5	5.6	89	0.5	48	Perimenopausal-Irregular	Right sided pelvic pain	Fig. 6
59		Positive	negative	negative	7.7	5.8	105.3	0.9	42	Follicular Phase	Fibroid uterus, abnormal uterine bleeding	Fig. 6

Tygerberg Hospital simultaneously test for HSV1&2 IgG, while Groote Schuur Hospital and Somerset Hospital performs individual tests for HSV1 IgG, HSV2 IgG and HSV IgM

Nd - Not Determined

Irregular as indicated by clerking sheet or lack of identifiable phase

Supplementary Table S3A: Repeated measures 2-way ANOVA for 100 nM MPA treated Ectocervical explants infected with HIV-1 BaLRenilla

Source of Variation	% of total variation	P value	P value summary	Significant?
Time	38.18	<0.0001	****	Yes
Treatment	1.335	0.1003	ns	No
Interaction: Time x Treatment	0.5175	0.113	ns	No

Sidak's multiple comparisons test	Mean Diff.	95.00% CI of diff.	Significant?	Summary	Adjusted P Value
Vehicle - 100 nM MPA					
0	0	-0.4939 to 0.4939	No	ns	>0.9999
3	-0.2621	-0.756 to 0.2318	No	ns	0.5959
5	-0.3862	-0.8801 to 0.1076	No	ns	0.196
7	-0.5581	-1.052 to -0.06419	Yes	*	0.0194
10	-0.6665	-1.16 to -0.1726	Yes	**	0.0033

Statistics performed on n = 19 due to missing time point (day 3) for one donor

Supplementary Table S3B: Repeated measures 2-way ANOVA for 10 nM MPA treated Ectocervical explants infected with HIV-1 BaLRenilla

Source of Variation	% of total variation	P value	P value summary	Significant?
Time	39.28	<0.0001	****	Yes
Treatment	0.0396	0.4896	ns	No
Interaction: time x Treatment	0.03516	0.634	ns	No

Sidak's multiple comparisons test	Mean Diff.	95.00% CI of diff.	Significant?	Summary	Adjusted P Value
Vehicle - 10 nM MPA					
0	0	-0.2319 to 0.2319	No	ns	>0.9999
3	-0.002037	-0.2339 to 0.2298	No	ns	>0.9999
5	0.06	-0.1719 to 0.2919	No	ns	0.9638
7	0.1509	-0.08094 to 0.3828	No	ns	0.3588
10	0.115	-0.1169 to 0.3469	No	ns	0.6408

Supplementary Table S3C: Repeated measures 2-way ANOVA for 1 nM MPA treated Ectocervical explants infected with HIV-1 BaLRenilla .

Source of Variation	% of total variation	P value	P value summary	Significant?
Time	50.18	0.0008	***	Yes
Treatment	0.2206	0.5064	ns	No
Interaction: Time x Treatment	0.172	0.6452	ns	No

Sidak's multiple comparisons test	Mean Diff.	95.00% CI of diff.	Significant?	Summary	Adjusted P Value
Vehicle - 1 nM MPA					
0	0	-0.6744 to 0.6744	No	ns	>0.9999
3	0.001333	-0.6731 to 0.6757	No	ns	>0.9999
5	0.2183	-0.4561 to 0.8927	No	ns	0.8926
7	0.2957	-0.3787 to 0.9701	No	ns	0.7115
10	0.4193	-0.2551 to 1.094	No	ns	0.3729

Supplementary Table S3D: Repeated measures 2-way ANOVA for 10 nM MPA treated Endocervical explants infected with HIV-1 BaLRenilla

Source of Variation	% of total variation	P value	P value summary	Significant?
Time	65.43	<0.0001	****	Yes
Treatment	1.633	0.1537	ns	No
Interaction: time x treatment	0.7645	0.1161	ns	No

Sidak's multiple comparisons test	Mean Diff.	95.00% CI of diff.	Significant?	Summary	Adjusted P Value
Vehicle - 10 nM MPA					
0	0	-0.6743 to 0.6743	No	ns	>0.9999
3	-0.295	-0.9693 to 0.3793	No	ns	0.7131
5	-0.365	-1.039 to 0.3093	No	ns	0.5147
7	-0.8383	-1.513 to -0.1641	Yes	*	0.0115
10	-0.7433	-1.418 to -0.06906	Yes	*	0.0271

Supplementary Table S4A: Repeated measures 2-way ANOVA for Ectocervical explants infected with HIV-1BaL-Renilla

Source of Variation	% of total variation	P value	P value summary	Significant?
Time	51.53	<0.0001	****	Yes
Treatment	1.713	0.0861	ns	No
Interaction: Time x Treatment	0.6922	0.007	**	Yes

Tukey's multiple comparisons test	Mean Diff.	95.00% CI of diff.	Significant?	Summary	Adjusted P Value
**Day 3**					
Vehicle vs. 100 nM MPA	-0.233	-0.5454 to 0.0795	No	ns	0.1814
Vehicle vs. 100 nM NET	0.003333	-0.3091 to 0.3158	No	ns	0.9996
100 nM MPA vs. 100 nM NET	0.2363	-0.07617 to 0.5488	No	ns	0.173
**Day 5**					
Vehicle vs. 100 nM MPA	-0.3756	-0.688 to -0.06309	Yes	*	0.0146
Vehicle vs. 100 nM NET	0.167	-0.1454 to 0.4795	No	ns	0.4101
100 nM MPA vs. 100 nM NET	0.5426	0.2301 to 0.8551	Yes	***	0.0003
**Day 7**					
Vehicle vs. 100 nM MPA	-0.4207	-0.7332 to -0.1083	Yes	**	0.0055
Vehicle vs. 100 nM NET	0.207	-0.1054 to 0.5195	No	ns	0.2574
100 nM MPA vs. 100 nM NET	0.6278	0.3153 to 0.9402	Yes	****	<0.0001
**Day 10**					
Vehicle vs. 100 nM MPA	-0.4756	-0.788 to -0.1631	Yes	**	0.0015
Vehicle vs. 100 nM NET	0.2944	-0.01802 to 0.6069	No	ns	0.0688
100 nM MPA vs. 100 nM NET	0.77	0.4575 to 1.082	Yes	****	<0.0001

Statistics performed on n = 9 due to missing time point (day 3) for one donor

Supplementary Table S4B: Repeated measures 2-way ANOVA for Endocervical explants infected with HIV-1 Bal-Renilla

Source of Variation	% of total variation	P value	P value summary	Significant?
Time	10.25	0.1639	ns	No
Treatment	1.705	0.3868	ns	No
Interaction: Time x Treatment	1.182	0.4305	ns	No

Tukey's multiple comparisons test	Mean Diff.	95.00% CI of diff.	Significant?	Summary	Adjusted P Value
**Day 3**					
Vehicle vs. 100 nM MPA	-1.121	-4.53 to 2.289	No	ns	0.7051
Vehicle vs. 100 nM NET	0.3183	-3.091 to 3.728	No	ns	0.972
100 nM MPA vs. 100 nM NET	1.439	-1.97 to 4.849	No	ns	0.5642
**Day 5**					
Vehicle vs. 100 nM MPA	-4.228	-7.638 to -0.8189	Yes	*	0.012
Vehicle vs. 100 nM NET	-0.6656	-4.075 to 2.744	No	ns	0.8834
100 nM MPA vs. 100 nM NET	3.563	0.1534 to 6.972	Yes	*	0.0389
**Day 7**					
Vehicle vs. 100 nM MPA	-2.585	-5.995 to 0.8241	No	ns	0.168
Vehicle vs. 100 nM NET	-2.473	-5.882 to 0.9369	No	ns	0.1943
100 nM MPA vs. 100 nM NET	0.1128	-3.297 to 3.522	No	ns	0.9964
**Day 10**					
Vehicle vs. 100 nM MPA	-2.807	-6.217 to 0.6022	No	ns	0.1243
Vehicle vs. 100 nM NET	-1.64	-5.05 to 1.769	No	ns	0.4771
100 nM MPA vs. 100 nM NET	1.167	-2.242 to 4.576	No	ns	0.6849

Supplementary Table S4C: Repeated measures 2-way ANOVA for Ectocervical explants infected with HIV-1 pNL4-3.

Source of Variation	% of total variation	P value	P value summary	Significant?
Time	28.88	0.0171	*	Yes
Treatment	2.196	0.1011	ns	No
Interaction: time x Treatment	1.264	0.0426	*	Yes

Tukey's multiple comparisons test	Mean Diff.	95.00% CI of diff.	Significant?	Summary	Adjusted P Value
**Day 3**					
Vehicle vs. 100 nM MPA	-0.08111	-0.6746 to 0.5124	No	ns	0.9395
Vehicle vs. 100 nM NET	0.2374	-0.3561 to 0.8309	No	ns	0.5911
100 nM MPA vs. 100 nM NET	0.3185	-0.275 to 0.912	No	ns	0.3937
**Day 5**					
Vehicle vs. 100 nM MPA	-0.1167	-0.9611 to 0.7277	No	ns	0.934
Vehicle vs. 100 nM NET	0.7583	-0.08607 to 1.603	No	ns	0.0828
100 nM MPA vs. 100 nM NET	0.875	0.0306 to 1.719	Yes	*	0.0416
**Day 7**					
Vehicle vs. 100 nM MPA	-0.1067	-0.7002 to 0.4868	No	ns	0.8978
Vehicle vs. 100 nM NET	0.8568	0.2633 to 1.45	Yes	**	0.0035
100 nM MPA vs. 100 nM NET	0.9635	0.37 to 1.557	Yes	**	0.0011
**Day 10**					
Vehicle vs. 100 nM MPA	-0.1633	-0.7568 to 0.4302	No	ns	0.7777
Vehicle vs. 100 nM NET	0.8466	0.2531 to 1.44	Yes	**	0.0039
100 nM MPA vs. 100 nM NET	1.01	0.4165 to 1.603	Yes	***	0.0006

Supplementary Table S5: Repeated measures 2-way ANOVA for 100 nM MPA +- RU486 treated ectocervical explants infected with HIV-1 BaLRenilla

Source of Variation	% of total variation	P value	P value summary	Significant?
Time	70.12	<0.0001	****	Yes
Treatment	3.975	0.3188	ns	No
Interaction: Time x Treatment	1.311	0.1862	ns	No

Tukey's multiple comparisons test	Mean Diff.	95.00% CI of diff.	Significant?	Summary	Adjusted P Value
**Day 3**					
Vehicle vs. 100 nM MPA	-0.3938	-0.7715 to -0.01604	Yes	*	0.0383
Vehicle vs. RU486	-0.04375	-0.4215 to 0.334	No	ns	0.9893
Vehicle vs. RU486/MPA	-0.03125	-0.409 to 0.3465	No	ns	0.996
100 nM MPA vs. RU486	0.35	-0.02771 to 0.7277	No	ns	0.0777
100 nM MPA vs. RU486/MPA	0.3625	-0.01521 to 0.7402	No	ns	0.0639
RU486 vs. RU486/MPA	0.0125	-0.3652 to 0.3902	No	ns	0.9997
**Day 5**					
Vehicle vs. 100 nM MPA	-0.4	-0.7777 to -0.02229	Yes	*	0.0345
Vehicle vs. RU486	0.0375	-0.3402 to 0.4152	No	ns	0.9932
Vehicle vs. RU486/MPA	0.09375	-0.284 to 0.4715	No	ns	0.9083
100 nM MPA vs. RU486	0.4375	0.05979 to 0.8152	Yes	*	0.0179
100 nM MPA vs. RU486/MPA	0.4938	0.116 to 0.8715	Yes	**	0.0062
RU486 vs. RU486/MPA	0.05625	-0.3215 to 0.434	No	ns	0.9778
**Day 7**					
Vehicle vs. 100 nM MPA	-0.3688	-0.7465 to 0.008959	No	ns	0.0578
Vehicle vs. RU486	0.175	-0.2027 to 0.5527	No	ns	0.6012
Vehicle vs. RU486/MPA	0.225	-0.1527 to 0.6027	No	ns	0.389
100 nM MPA vs. RU486	0.5438	0.166 to 0.9215	Yes	**	0.0023
100 nM MPA vs. RU486/MPA	0.5938	0.216 to 0.9715	Yes	***	0.0008
RU486 vs. RU486/MPA	0.05	-0.3277 to 0.4277	No	ns	0.9842
**Day 10**					
Vehicle vs. 100 nM MPA	-0.3375	-0.7152 to 0.04021	No	ns	0.0939
Vehicle vs. RU486	0.1688	-0.209 to 0.5465	No	ns	0.6288
Vehicle vs. RU486/MPA	0.2875	-0.09021 to 0.6652	No	ns	0.1891
100 nM MPA vs. RU486	0.5063	0.1285 to 0.884	Yes	**	0.0049
100 nM MPA vs. RU486/MPA	0.625	0.2473 to 1.003	Yes	***	0.0004
RU486 vs. RU486/MPA	0.1188	-0.259 to 0.4965	No	ns	0.8318

Supplementary Table S6. Mean and standard deviations of observed protein concentrations determined by Luminex or ELISA

Soluble mediator	Dose	Treatment	Mean pg/mL	Standard Deviation
**Eaotaxin[(1)]**	100 nM	Vehicle	39.81	25
		MPA	45.16	31.72
		NET	34.43	25.07
	10 nM	Vehicle	32.71	22.83
		MPA	30.22	20.09
**IL-1RA [(1)]**	100 nM	Vehicle	4414	5913
		MPA	3090	1800
		NET	4265	4479
	10 nM	Vehicle	1619	2573
		MPA	620.2	626.5
**IL6 [(1)]**	100 nM	Vehicle	2038	3361
		MPA	2234	3624
		NET	6206	12802
	10 nM	Vehicle	515.6	1065
		MPA	1832	1986
**IL-6 [(2)]**	100 nM	Vehicle	2622	1699
		MPA	2480	1574
		NET	2503	1717
**IL8 [(1)]**	100 nM	Vehicle Vehicle	8187 7776 4415	4475
		MPA	7621	4372
		NET	7287	4623
	10 nM	Vehicle	7836	3899
		MPA	7899	4697
**IL-8 [(2)]**	100 nM	Vehicle	8755	4049
		MPA	8269	3786
		NET	7733	4800
**MCP-1 [(1)]**	100 nM	Vehicle	2870	2104
		MPA	2639	2077
		NET	2617	2291
	10 nM	Vehicle	2935	2077
		MPA	3043	2545
**RANTES [(1)]**	100 nM	Vehicle	107.8	51.95
		MPA	95.62	49.66
		NET	107.9	61.11
	10 nM	Vehicle	46.43	41.18
		MPA	53.96	48.61
**SLPI [(1)]**	100 nM	Vehicle	7123	6310
		MPA	5800	5642
		NET	9248	7693
	10 nM	Vehicle	15345	17106
		MPA	18093	18154
**SLP1 [(2)]**	100 nM	Vehicle	8301	6807
		MPA	7600	5967
		NET	6363	8314

Mean concentration (pg/mL) and standard deviation of the soluble mediator as measured by (1) Luminex or (2) ELISA.

Supplementary Table S7: % viability of ungated population following 48 hr and 7 days post treatment

**48 hr**	**Vehicle**		**MPA**		**NET**	
	**%viable**	**% dead**	**%viable**	**% dead**	**%viable**	**% dead**
**Donor 34**	97.6	2.36	97.7	2.31	96.3	3.72
**Donor 35**	94.8	5.22	98.7	1.29	99.4	0.64
**Donor 38**	73	27	65.1	34.9	72.2	27.8
**Mean**	88.47	11.53	87.17	12.83	89.30	10.72
**SD**	13.47	13.48	19.12	19.12	14.89	14.87
	**Vehicle**		**MPA**		**NET**	
**Day 7**	**%viable**	**% dead**	**%viable**	**% dead**	**%viable**	**% dead**
**Donor 40**	97.1	2.88	98.6	1.36	98.7	1.35
**Donor 41**	99.1	0.92	98	1.96	94.8	5.24
**Donor 42**	99.9	0.057	99.4	0.61	99.8	0.2
**Donor 43**	99.6	0.41	99.6	0.44	99.6	0.4
**Donor 44**	99.1	0.87	99.2	0.79	98.1	1.9
**Donor 45**	99.5	0.54	94.3	5.75	99.8	0.22
**Donor 46**	97.7	2.3	94.3	5.68	99.3	0.7
**Donor 47**	99.7	0.3	99.1	0.87	99.4	0.64
**Mean**	98.96	1.03	97.81	2.18	98.69	1.33
**SD**	1.02	1.01	2.22	2.23	1.68	1.68

Supplementary Table S8A. Frequencies of T cells and monocytes expressing CD69 or CCR5 in ectocervical explants stimulated with 100 nM MPA or NET versus control for 48 hr

Immune cell phenotype	Vehicle	100 nM MPA	100 nM NET
**CD3+ % total**	42.53 (14.5)	36.8 (8.764)	26.73 (11.230)
**CD3+CCR5+**	97.83 (1.084)	97.2 (1.904)	98.63 (0.367)
**CD3+CD69+**	37.03 (12.15)	40.57 (16.69)	37.67 (15.39)
**CD3+CD69+CCR5+**	99.3 (0.436)	98.73 (0.819)	98.97 (0.176)
**CD4+ % total**	24.29 (11.57)	26.88 (14.2)	17.96 (6.794)
**CD4+CCR5+**	96.53 (1.943)	97 (1.29)	97.93 (0.561)
**CD4+CD69+**	48.03 (13.48)	50.8 (18.06)	53.4 (14.46)
**CD4+CD69+CCR5+**	97.87(1.749)	97.7 (1.15)	98.46 (0.841)
**CD8+ % total**	36.83 (5.63)	35.3 (2.954)	33.2 (4051)
**CD8+CCR5+**	99.27 (0.41)	99.1 (0.5)	99.33 (0.12)
**CD8+CD69+**	36.13 (11.13)	39.43 (15.71)	37.76 (16.93)
**CD8+CD69+CCR5+**	99.76 (0.12)	99.43 (0.47)	99.1 (0.361)
**CD14+ % total**	56.23 (16.41)	60.9 (22.52)	39.86 (12.53)
**CD14+CCR5+**	96.16 (0.376)	96.67 (0.318)	92.3 (2.495)
**CD14+CD69+**	16.64 (6.005)	13.87 (7.981)	11.48 (5.123)
**CD14+CD69+CCR5+**	99.33 (0.441)	96.33 (2.717)	98.13 (1.065)
**CD4+/CD8+ ratio**	0.64 (0.311)	0.84 (0.507)	0.504 (0.153)

Average frequency (±SEM) indicated for n=3 independent explant donors. Statistical comparisons were carried between conditions out using parametric one-way ANOVA with Tukey’s post-test or non-parametric Kruskal-Wallis with Dunn’s post-test.

Supplementary Table S8B. Densities of cells expressing CD69 or CCR5 in ectocervical explants stimulated with 100 nM MPA or NET versus control for 48 hr

Immune cell phenotype	Vehicle	100 nM MPA	100 nM NET
**CD3+CCR5+ MFI**	29112 (8718)	24794 (7624)	25724.33 (5849)
**CD3+CD69+MFI**	15887.67 (8123)	16028.33 (8174)	16709.67 (8594)
**CD3+CD69+CCR5+ (CCR5 MFI)**	30711.67 (9163)	27288.66 (8929)	28534.33 (6801)
**CD3+CD69+CCR5+ (CD69 MFI)**	15884.33 (8126)	16037 (8171)	16709.67 (8594)
**CD4+CCR5+ MFI**	28819.33 (8880)	26576.33 (8974)	26467 (5604)
**CD4+CD69+MFI**	17890.67 (8647)	16957.33 (7961)	17637.67 (7995)
**CD4+CD69+CCR5+ (CCR5 MFI)**	32268.67 (10007)	30073.67 (10502)	31838 (8834)
**CD4+CD69+CCR5+ (CD69 MFI)**	17937.67 (8616)	16998.33 (7940)	17652 (7989)
**CD8+CCR5+ MFI**	26738 (7906)	22847 (5789)	24259 (5604)
**CD8+CD69+MFI**	15400 (8082)	15326.33 (8011)	16342 (8934)
**CD8+CD69+CCR5+ (CCR5 MFI)**	27791.33 (8434)	25732.67 (7721)	25206.33 (5816)
**CD8+CD69+CCR5+ (CD69 MFI)**	15400 (8082)	15326.33 (8011)	16343 (8932)
**CD14+CCR5+ MFI**	61244 (36253)	32539.67 (12817)	31581.33 (10061)
**CD14+CD69+MFI**	6688.67 (2967)	6243 (2369)	6674.67 (2599)
**CD14+CD69+CCR5+ (CCR5 MFI)**	94254.67 (53090)	53462 (28496)	42570.67 (10660)
**CD14+CD69+CCR5+ (CD69 MFI)**	6688.33 (2967)	6243 (2369)	6804.33(2712)

Average median fluorescent intensity (MFI) ±SEM indicated for n=3 independent explant donors. Statistical comparisons were carried between conditions out using parametric one-way ANOVA with Tukey’s post-test or non-parametric Kruskal-Wallis with Dunn’s post-test.

Supplementary Table S9A. Frequencies of T cells and monocytes expressing CD69 or CCR5 in ectocervical explants stimulated with 100 nM MPA or NET versus control for 7 days

Immune cell phenotype	Vehicle	100 nM MPA	100 nM NET
**CD3+ % total**	31.24 (10.28)	38.85 (6.746)	31.91 (10.3)
**CD3+CCR5+**	77.1 (3.181)	83.48 (2.923)	78.21 (5.402)
**CD3+CD69+**	25.11 (4.957)	25.8 (4.237)	26.66 (6.348)
**CD3+CD69+CCR5+**	88.6 (3.337)	91.76 (1.991)	91.19 (1.995)
**CD4+ % total**	2.804 (1.127)	**4.349 (1.743)**	5.26 (2.47)
**CD4+CCR5+**	87.71(4.456)	89.13 (5.041)	90.58 (2.166)
**CD4+CD69+**	68.94 (6.257)	57.33(4.137)	62.14 (6.939)
**CD4+CD69+CCR5+**	91.04 (4.894)	92.65(4.99)	91.73 (2.467)
**CD8+ % total**	22.03 (5.091)	23.91 (3.823)	24.3 (6.617)
**CD8+CCR5+**	87.26 (2.874)	91.78 (1.924)	88.84 (2.81)
**CD8+CD69+**	55.2 (3.658)	54.18 (5.047)	59.01 (3.206)
**CD8+CD69+CCR5+**	88.99 (3.431)	94.16 (2.062)	91.78(2.884)
**CD14+ % total**	20.62 (6.31)	30.92 (5.45)	19.67 (5.349)
**CD14+CCR5+**	97.43 (1.064)	98.28 (0.4898)	97.55 (0.9194)
**CD14+CD69+**	13.74 (2.388)	**18.92 (2.761)**	15.25 (2.58)
**CD14+CD69+CCR5+**	99.05 (0.3742)	99.56 (0.1647)	99.49 (0.2649)
**CD4/CD8 ratio**	0.1556 (0.06186)	0.1944 (0.06786)	0.2175 (0.07601)

Average frequency (±SEM) indicated for n=8 independent explant donors. Statistical comparisons were carried between conditions out using parametric one-way ANOVA with Tukey's post-test or non-parametric Kruskal-Wallis with Dunn's post-test. **Bold** indicates fold change significance, as shown in Figure 7.

Supplementary Table S9B. Densities of cells expressing CD69 or CCR5 in ectocervical explants stimulated with 100 nM MPA or NET versus control for 7 days

Immune cell phenotype	Vehicle	100 nM MPA	100 nM NET
**CD3+CCR5+ MFI**	7114 (452.1)	7376 (468.9)	7778 (674.4)
**CD3+CD69+MFI**	3572 (370.9)	3639 (418.6)	3726 (340.4)
**CD3+CD69+CCR5+ (CCR5 MFI)**	9194 (971)	9268 (878.1)	9766 (992.3)
**CD3+CD69+CCR5+ (CD69 MFI)**	3531 (365.5)	3639 (430.7)	3703 (342.6)
**CD4+CCR5+ MFI**	8798 (1003)	10190 (1635)	12808 (2053)
**CD4+CD69+MFI**	3705 (602.5)	3757 (500.3)	4370 (543.6)
**CD4+CD69+CCR5+ (CCR5 MFI)**	9265(1054)	10635 (1829)	13251 (2097)
**CD4+CD69+CCR5+ (CD69 MFI)**	3728 (593.6)	3794 (521.5)	437 (584)
**CD8+CCR5+ MFI**	8076(714.7)	8520 (781.4)	8616 (923.4)
**CD8+CD69+MFI**	4061 (312.8)	4127 (422.1)	4299 (289.5)
**CD8+CD69+CCR5+ (CCR5 MFI)**	8679 (876.4)	9057 (944.5)	9142 (1020)
**CD8+CD69+CCR5+ (CD69 MFI)**	4026 (297.5)	4108 (420.9	4260 (262.6)
**CD14+CCR5+ MFI**	12685 (1316)	13583 (1277)	13798 (1203)
**CD14+CD69+MFI**	2785 (177.8)	2946 (189.3)	2907 (190.3)
**CD14+CD69+CCR5+ (CCR5 MFI)**	17551 (2094)	20019 (2445)	19944 (1995)
**CD14+CD69+CCR5+ (CD69 MFI)**	2782 (177.3)	2944 (189.2)	2901 (189.9)

Average median fluorescent intensity (MFI) ±SEM indicated for n=8 independent explant donors. Statistical comparisons were carried between conditions out using parametric one-way ANOVA with Tukey's post-test or non-parametric Kruskal-Wallis with Dunn's post-test.

Supplementary Table S10: Absolute cell number of CD4 and CD4CCR5+ cells of individual donors

Absolute cell number	CD4+			CD4+CCR5+		
**48 hr**						
	**Vehicle**	**100 nM MPA**	**100 nM NET**	**Vehicle**	**100 nM MPA**	**100 nM NET**
**Donor 34**	926	582	824	897	554	805
**Donor 35**	666	408	197	664	406	195
**Donor 38**	993	572	583	923	551	566
**Mean (±SD)**	861.7 (172.7)	520.7 (97.7)	534.7 (316.3)	828 (142.6)	503.7 (84.6)	522 (307.4)
**Day 7**						
**Donor 40**	256	605	252	216	454	135
**Donor 41**	25	143	67	23	138	65
**Donor 42**	8	144	15	7	144	14
**Donor 43**	660	632	973	515	514	806
**Donor 44**	488	728	757	423	617	682
**Donor 45**	170	2126	95	165	2078	84
**Donor 46**	946	5817	2683	924	5676	2643
**Donor 47**	174	319	165	159	296	155
**Mean (±SD)**	340.9 (330.5)	1314 (1926)	625.9 (901.5)	304 (306.7)	1240 (1898)	573 (888.6)

Absolute cell number of CD4+ and CD4+CCR5+ cells of individual donors at 48 hr (n=3) and 7 days (n=8) post stimulation as measured by flow cytometry on gated viable CD3+ cells (as shown in gating strategy in figure 3S). Mean (±SD) of donors 48 hr (n=3) and 7 days (n=8) post stimulation are also indicated.

Supplementary Table S11: MFI of CD4 cells of individual donors

	CD4 MFI		
**48 hr**	**Vehicle**	**100 nM MPA**	**100 nM NET**
**Donor 34**	5640	5359	5750
**Donor 35**	4481	4986	5169
**Donor 38**	3518	3208	3693
**Mean (±SD)**	4546.33 (1062.5)	4517.66 (1149.43)	4870.66 (1060.45)
**Day 7**			
**Donor 40**	3539	3525	3372
**Donor 41**	6733	6432	5652
**Donor 42**	7253	6631	7414
**Donor 43**	3539	3800	3902
**Donor 44**	5093	4024	5268
**Donor 45**	4008	3934	4566
**Donor 46**	2609	2702	3590
**Donor 47**	2928	3132	2815
**Mean (±SD)**	4462.75 (1733.78)	4272.5 (1461.48)	4572.37 (1496.09)

Notes: MFI: Median fluorescent Intensity

Supplementary Figures

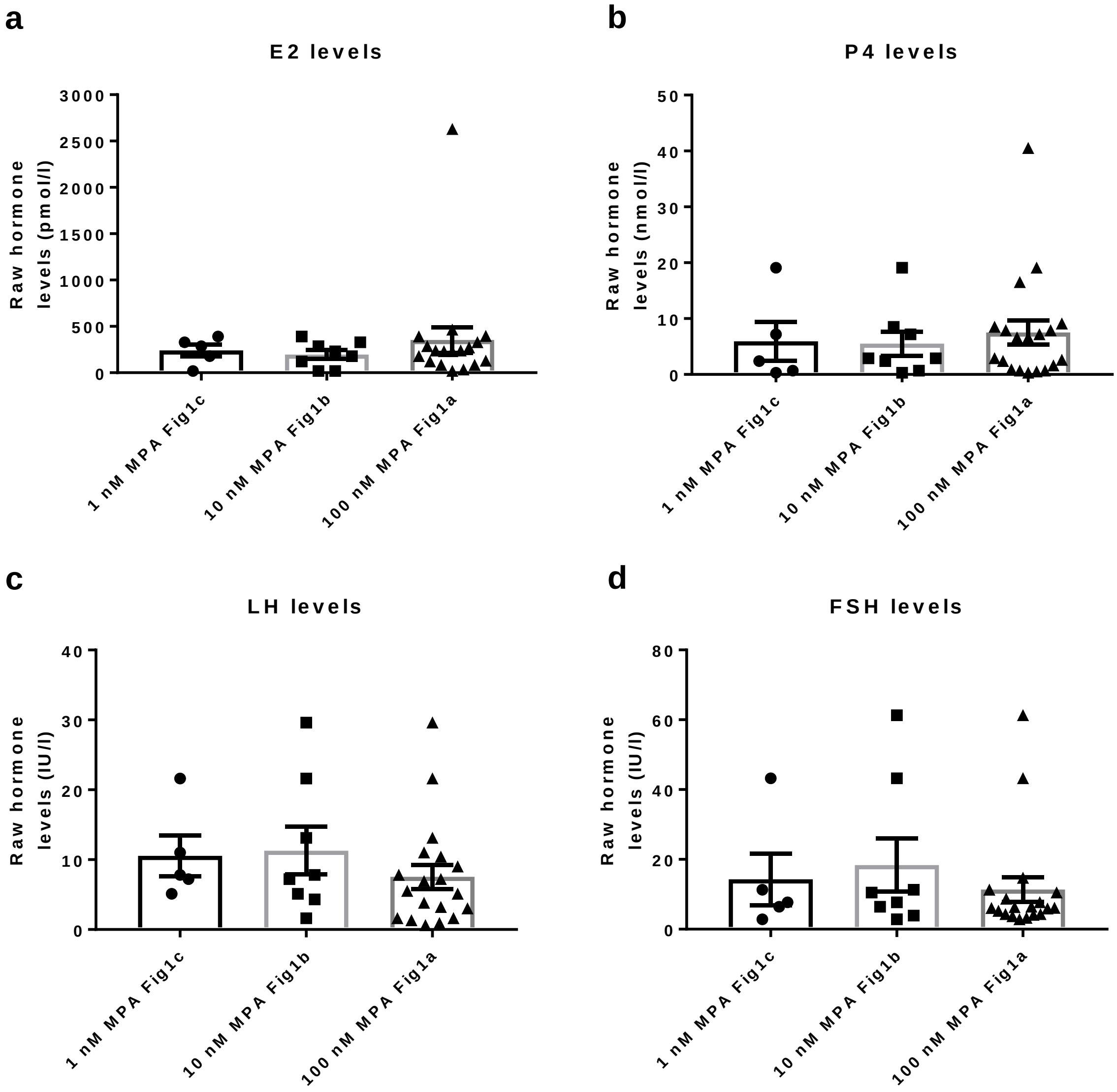

Figure S1: Donor endogenous hormone levels are not significantly different.

Endogenous E2 (a), P4 (b), LH (c) and FSH (d) hormone levels available for the individual donors used in tissue infection experiments from Figure 1 (a-c) were pooled. For two of the donors used in Figure 1 (a-c), the hormone levels are not known and they have been excluded from this graph. Histograms show mean ± SEM of n = 5 for 1 nM MPA, n = 8 for 10 nM MPA and n = 19 for 100 nM MPA.

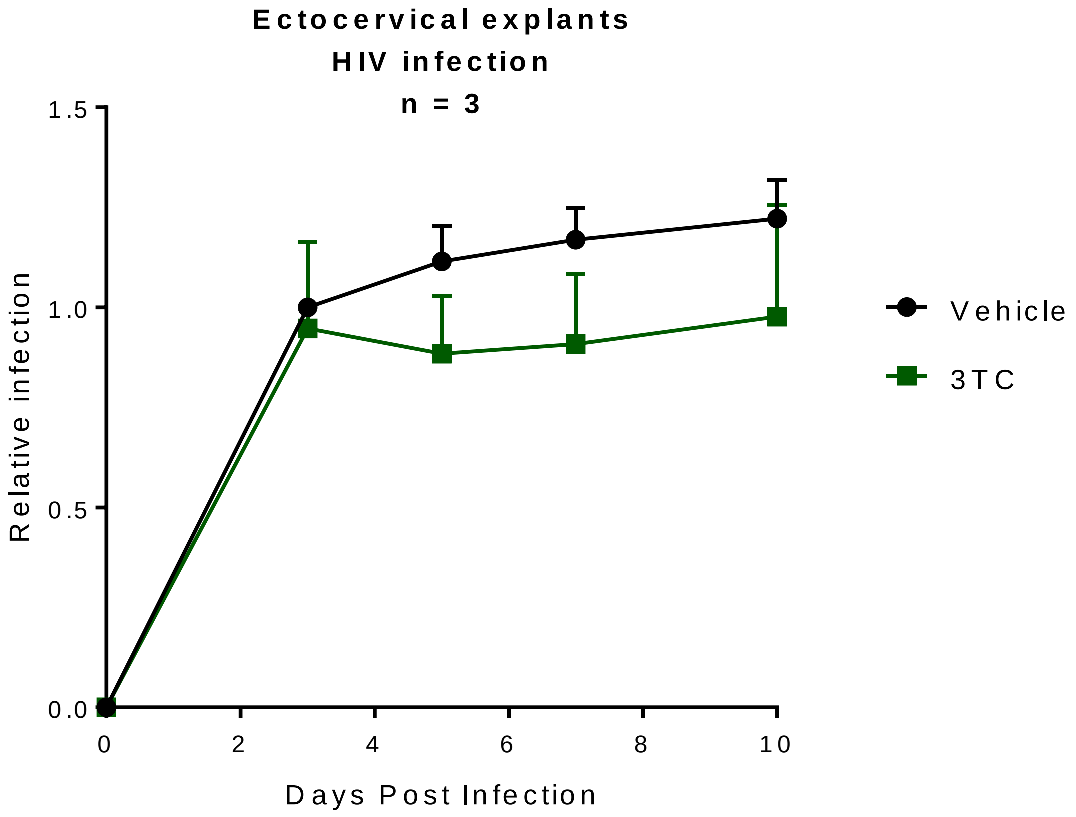

Figure S2: Ectocervical explants are productively infected with HIV-1.

Explants were treated in parallel with vehicle (DMSO (0.1% v/v)) or 5 (n = 1) or 10 µM (n = 2) 3TC for 1 (n = 2) or 48 hr (n = 1) before adding 1000 (n = 2) or 10000 (n = 1) IU/mL HIV-1 IMCs and incubating for a further 2 hr. Thereafter explants were washed several times with 1 X PBS and added back to fresh media. Half of the supernatants were collected every 2 – 3 days and media were replaced in the presence of 3TC. The variations in time of preincubation with 3TC, [3TC] and infectious units of HIV-1 as indicated did not detectably change the results. Relative infection was determined by p24 ELISA analysis, and normalized by setting the day 3 vehicle to 1. Graphs show cumulative curve of p24 production over time (from days 0, 3, 5, 7 and 10 post HIV infection). Each condition was performed in triplicate and pooled data is shown for three independent experiments with three different donors, with XY plots showing mean ± SEM.

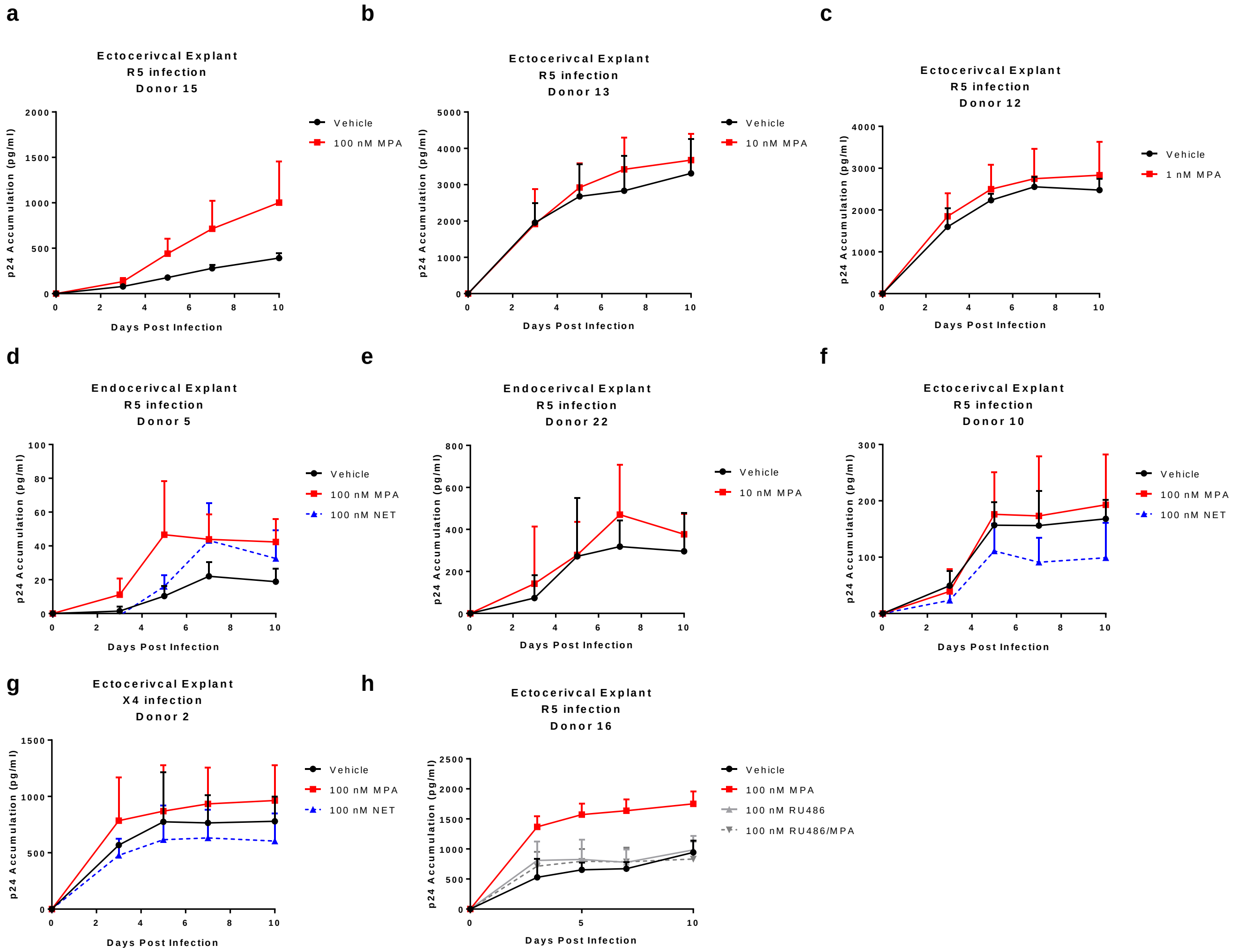
a
Ectocerivcal Explant
R5 infection
Donor 15
p24 Accumulation (pg/ml)
Days Post Infection
Vehicle
100 nM MPA
b
Ectocerivcal Explant
R5 infection
Donor 13
p24 Accumulation (pg/ml)
Days Post Infection
Vehicle
10 nM MPA
c
Ectocerivcal Explant
R5 infection
Donor 12
p24 Accumulation (pg/ml)
Days Post Infection
Vehicle
1 nM MPA
d
Endocerivcal Explant
R5 infection
Donor 5
p24 Accumulation (pg/ml)
Days Post Infection
Vehicle
100 nM MPA
100 nM NET
e
Endocerivcal Explant
R5 infection
Donor 22
p24 Accumulation (pg/ml)
Days Post Infection
Vehicle
10 nM MPA
f
Ectocerivcal Explant
R5 infection
Donor 10
p24 Accumulation (pg/ml)
Days Post Infection
Vehicle
100 nM MPA
100 nM NET
g
Ectocerivcal Explant
X4 infection
Donor 2
p24 Accumulation (pg/ml)
Days Post Infection
Vehicle
100 nM MPA
100 nM NET
h
Ectocerivcal Explant
R5 infection
Donor 16
p24 Accumulation (pg/ml)
Days Post Infection
Vehicle
100 nM MPA
100 nM RU486
100 nM RU486/MPA

Figure S3: p24 accumulation curves over time for individual representative donor cervical explants. Representative p24 infection data (pg/mL) are shown for individual donor tissue infection experiments from Figures 1 - 3. Explants were pre-treated in parallel for 48 hr in parallel with vehicle or progestins as indicated in Figures 1 - 3 and graphs show p24 accumulation at time points 0, 3, 5, 7 and 10 days post infection. Explants were infected with either HIV-1$_{\text{BaL-Renilla}}$ (a,c,d-h) or HIV-1$_{\text{PNL4.3}}$ (b). Each condition was performed at least in triplicate. Data is representative of individual donors, with XY plots showing mean + SD.

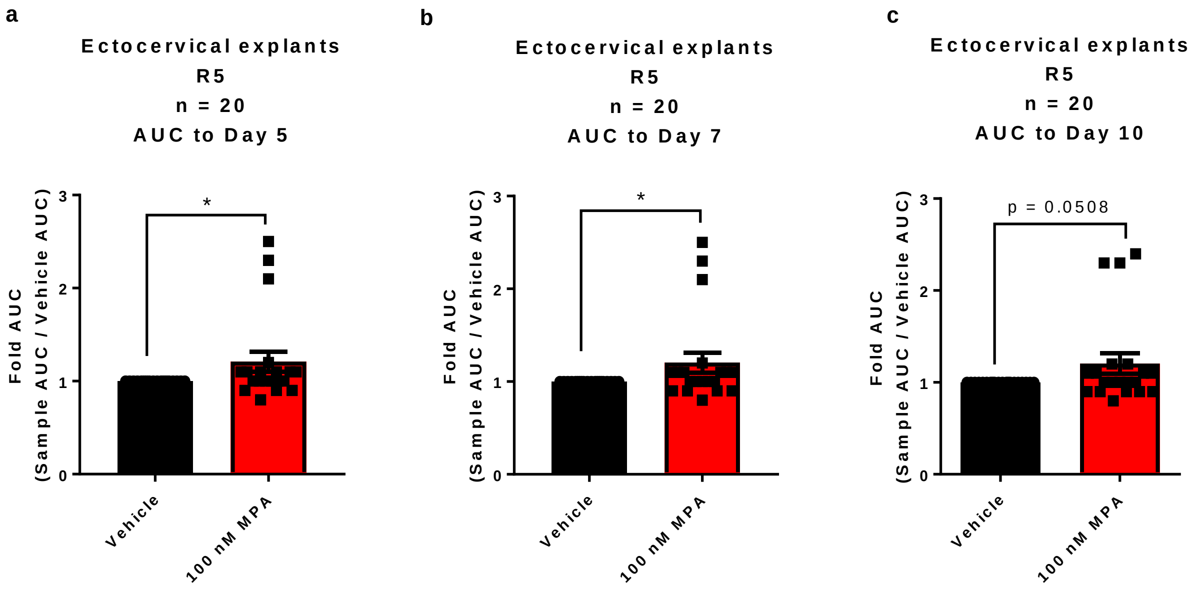

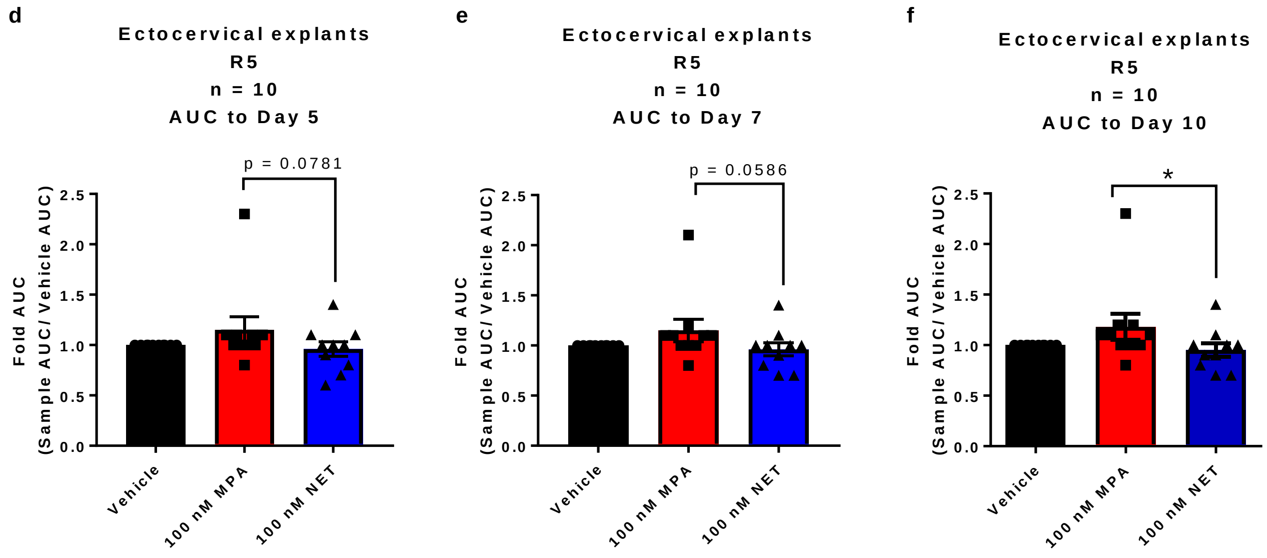

Figure S4: Area under the curve analyses were performed on R5 infected ectocervical explants treated with (a-c) 100 nM MPA or vehicle control as well as R5 infected ectocervical samples treated with (d-f) 100 nM MPA or NET. AUCs were calculated over different time points with (a and d) AUC up to Day 5, (b and e) up to Day 7 and (c and f) up to Day 10 post infection. AUCs were calculated using Graphpad Prism software version 6, with AUCs normalized to the vehicle control set to 1. Histograms are representative of mean ± SEM. For a-c, non-parametric paired Wilcoxon signed rank tests were performed on each of the data sets with p = 0.00179 for Day 5, p = 0.0258 for Day 7 and p = 0.0508 for Day 10. For (f), a non-parametric Friedman one-way ANOVA was performed with a post-hoc Dunn's test for multiple comparisons with p = 0.0417 for MPA vs NET. For (d) and (e) a non-parametric paired Wilcoxon signed rank tests were performed with p = 0.0781 (d) and p = 0.0586 (e).

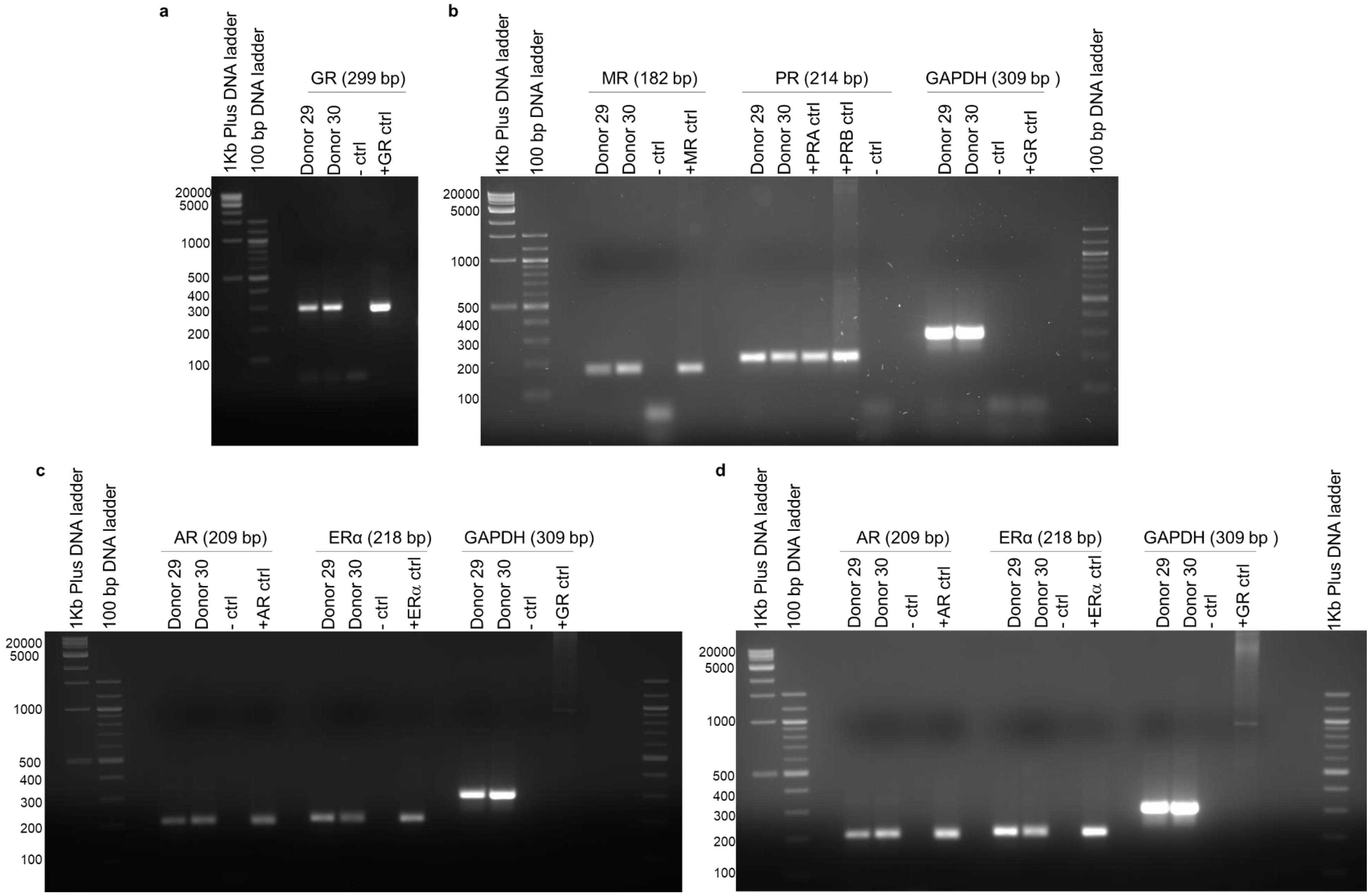
a
1Kb Plus DNA ladder
100 bp DNA ladder
GR (299 bp)
Donor 29
Donor 30
- ctrl
+GR ctrl
20000
5000
1000
500
400
300
200
100
b
1Kb Plus DNA ladder
100 bp DNA ladder
MR (182 bp)
Donor 29
Donor 30
- ctrl
+MR ctrl
PR (214 bp)
Donor 29
Donor 30
+PRA ctrl
+PRB ctrl
- ctrl
GAPDH (309 bp)
Donor 29
Donor 30
- ctrl
+GR ctrl
100 bp DNA ladder
20000
5000
1000
500
400
300
200
100
c
1Kb Plus DNA ladder
100 bp DNA ladder
AR (209 bp)
Donor 29
Donor 30
- ctrl
+AR ctrl
ERα (218 bp)
Donor 29
Donor 30
- ctrl
+ERα ctrl
GAPDH (309 bp)
Donor 29
Donor 30
- ctrl
+GR ctrl
20000
5000
1000
500
400
300
200
100
d
1Kb Plus DNA ladder
100 bp DNA ladder
AR (209 bp)
Donor 29
Donor 30
- ctrl
+AR ctrl
ERα (218 bp)
Donor 29
Donor 30
- ctrl
+ERα ctrl
GAPDH (309 bp)
Donor 29
Donor 30
- ctrl
+GR ctrl
1Kb Plus DNA ladder
20000
5000
1000
500
400
300
200
100

Figure S5. Steroid receptor mRNA levels showing separate gels and exposures used for Figure 3. Total RNA from two donors was analysed for the different steroid receptor mRNAs by RT-PCR. RT-PCR products were electrophoresis on an agarose gel stained with ethidium bromide and products visualised under UV for steroid receptor mRNA (see methods for details). Separate gels were used for electrophoreses of (a) GR products (1 min exposure) (b) MR and PR and GAPDH products (3 mins exposure) and (c-d) AR and ERα and GAPDH products with two exposures of (c) 1 min and (d) 3 mins. Sizes were determined by comparison to the 1 Kb plus and 100 bp DNA ladders were as indicated. For presentation purposes in Figure 3A, products were cropped out of respective gels and arranged in order of specific products of Donor 29 and 30, -ctrl (H20) and + ctrl (positive steroid receptor control plasmid). GR products and MR products were cropped from the gel a, b. The PR products were cropped from gel b and rearranged to fit the presentation style. GAPDH products were cropped from gel c to avoid overexposure of GAPDH and AR and ERα products were cropped from d.

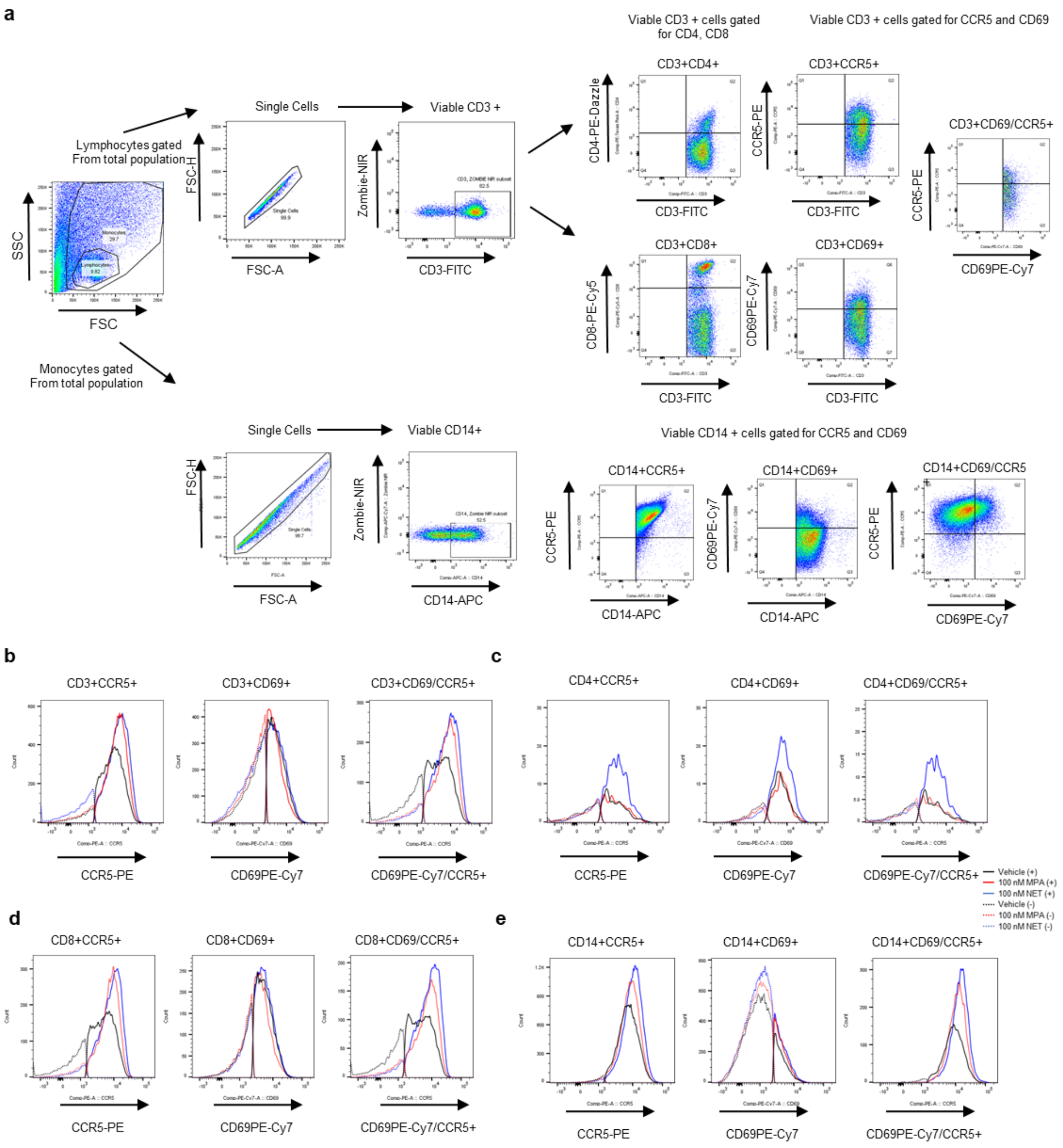

Figure S6. Representative gating strategy for analysis of viable lymphocytes and monocytes isolated from ectocervical explants 48 hr and 7 days post treatment. Ectocervical explants were treated in parallel with 100 nM MPA or NET or a vehicle control (EtOH) for 48 hr or 7 days followed by tissue digestion. Thereafter cells were stained for different cell surface receptors and analysed by flow cytometry. Graphs show the gating

strategy of one representative donor at day 7 post treatment. (a) Monocytes and lymphocytes were gated from the total cellular population, after which only the single cell population was used for analysis. Viable CD3+ cells were gated using the ZOMBIE NIR viability stain and CD3 marker. For lymphocytes, CD4 and CD8 cells were gated from the viable CD3+ population and CCR5 and CD69 cells using the appropriate MFO controls. For monocytes, viable CD14+ cells were used and CCR5+ and CD69+ expression levels were determined using the appropriate MFO controls. (b-e) Representative histogram distributions of CCR5+, CD69+ or CD69+ CCR5+ expressing (b) CD3+ cells, (c) CD4+ cells, (d) CD8+ cells, (e) CD14+ cells following 7-day incubation with ligands. Solid line indicates + population, dashed line indicates negative population based on MFO.

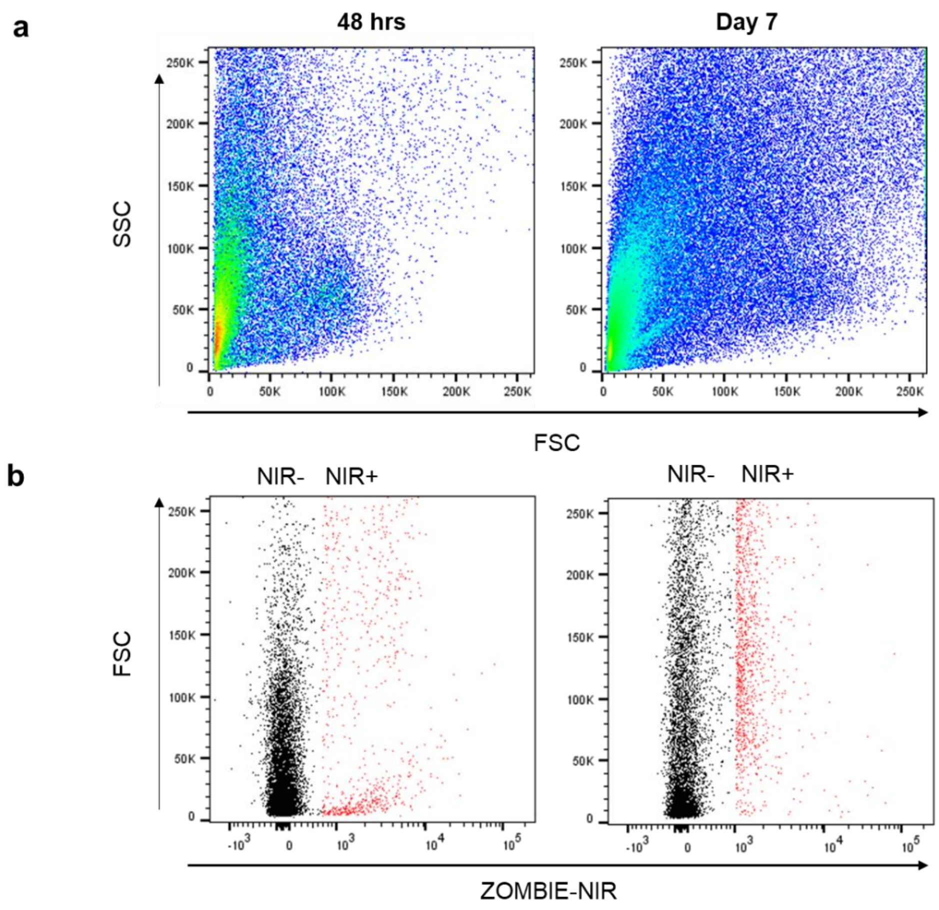

Figure S7. Lymphocytes acquired after a tissue digestion of ectocervical explants are viable post treatment.

Ectocervical explant tissue were stimulated in parallel with vehicle (EtOH) or 100 nM MPA or 100 nM NET for 48 hr or 7 days after which tissue was digested and processed for flow cytometry analysis. (a) Representative ungated FSC vs SSC donor dot plots after 48 hr and 7 days are shown. (b) Viability analysis done on ungated populations to assess the % viability of cells. Representative donor dot plots showing viability analysis 48 hr and Day 7 post vehicle stimulation on ungated populations using the ZOMBIE NIR viability stain. The % viability of ungated population was calculated and indicated in supplementary Table S7.

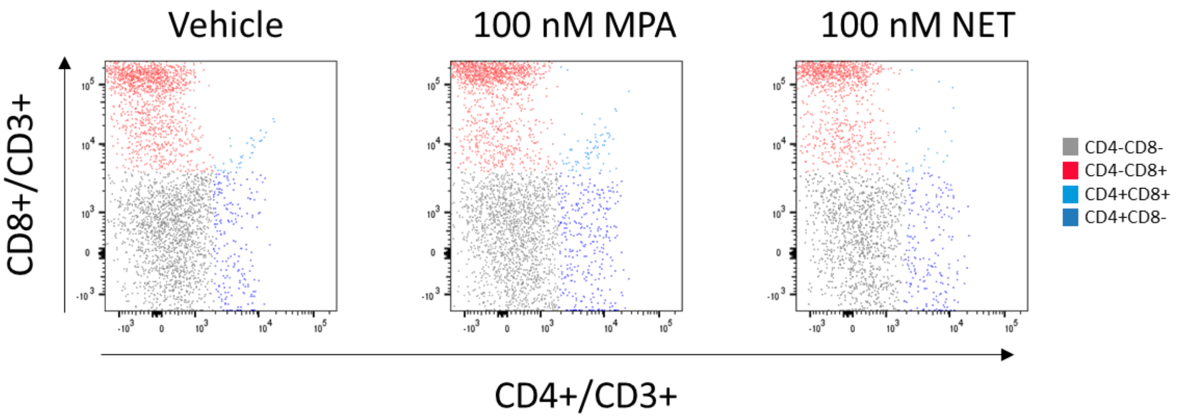

Figure S8. Representative scatter plots of CD8 and CD4 scatter plot of CD3+ cells of the 3 treatment groups at day 7.

Ectocervical explant tissue were stimulated in parallel with vehicle (EtOH) or 100 nM MPA or 100 nM NET for 7 days after which the frequency of CD4+ and CD8+ of live CD3+ cells were determined using flow cytometry.
